# Structure-guided product determination of the bacterial type II diterpene synthase Tpn2

**DOI:** 10.1038/s42004-022-00765-6

**Published:** 2022-11-08

**Authors:** Emma A. Stowell, Michelle A. Ehrenberger, Ya-Lin Lin, Chin-Yuan Chang, Jeffrey D. Rudolf

**Affiliations:** 1 Department of Chemistry, University of Florida, Gainesville, FL 32611 USA; 2 Department of Biological Science and Technology, National Yang Ming Chiao Tung University, Hsinchu, 30010 Taiwan, ROC; 3 Center for Intelligent Drug Systems and Smart Bio-devices, National Yang Ming Chiao Tung University, Hsinchu, 30010 Taiwan, ROC

**Keywords:** Enzyme mechanisms, X-ray crystallography, Biosynthesis, Natural products

## Abstract

A grand challenge in terpene synthase (TS) enzymology is the ability to predict function from protein sequence. Given the limited number of characterized bacterial TSs and significant sequence diversities between them and their eukaryotic counterparts, this is currently impossible. To contribute towards understanding the sequence-structure-function relationships of type II bacterial TSs, we determined the structure of the terpentedienyl diphosphate synthase Tpn2 from *Kitasatospora* sp. CB02891 by X-ray crystallography and made structure-guided mutants to probe its mechanism. Substitution of a glycine into a basic residue changed the product preference from the clerodane skeleton to a *syn-*labdane skeleton, resulting in the first *syn*-labdane identified from a bacterial TS. Understanding how a single residue can dictate the cyclization pattern in Tpn2, along with detailed bioinformatics analysis of bacterial type II TSs, sets the stage for the investigation of the functional scope of bacterial type II TSs and the discovery of novel bacterial terpenoids.

## Abstract (web summary)

Crystal structure elucidation of terpene synthases plays an important role to understand the sequence-structure-function relationships of this enzyme class. Here, the authors determine the crystal structure of bacterial terpene synthase Tpn2, probe the catalytic mechanism by structure-guided mutation, and change the product preference from a clerodane skeleton to a *syn*-labdane skeleton.

## Introduction

Terpenoids are a large family of natural products biosynthesized from five-carbon building blocks. Acyclic precursors, such as the universal precursor for the C_20_ diterpenes geranylgeranyl diphosphate (GGPP, **1**), are often cyclized by terpene synthases (TSs) and the resulting mono- or polycyclic hydrocarbon skeletons are subsequently modified by a variety of tailoring enzymes leading to the extraordinary diversity of structures and biological functions seen in this class of natural products^1–3^. Terpenoids are one of the largest and most diverse families of natural products, with over 84,000 members currently characterized^4^. However, terpenoids of bacterial origin represent only a small fraction (<2%) of this family of natural products^1^. For example, there are over 2300 clerodane natural products known in nature, but only four are from bacteria^4–7^.

TSs employ carbocation chemistry to convert acyclic prenyl diphosphates into diverse terpene skeletons. These reactions are quite complex, frequently involving changes in bonding, hybridization, or configuration for most the carbons in the substrate through a variety of reactions including carbon-carbon bond formation, hydride and alkyl shifts, and eliminations^8^. The conformational flexibility of terpene substrates paired with the inherent reactivity of each cationic intermediate allows TSs to provide a diverse array of molecular templates for folding the substrate into catalytically relevant poses and stabilizing intermediates^9–11^. Canonical bacterial TSs are categorized into type I or type II depending on how they initiate catalysis. Type I TSs, which have conserved metal ion-binding motifs DDxxD and NSE/DTE, abstract the diphosphate group from the substrate^8,12–14^. Type II TSs utilize a highly conserved DxDD motif, in which the central Asp residue acts as a general acid, to protonate an alkene or epoxide^8,15^.

**Fig. 1:**
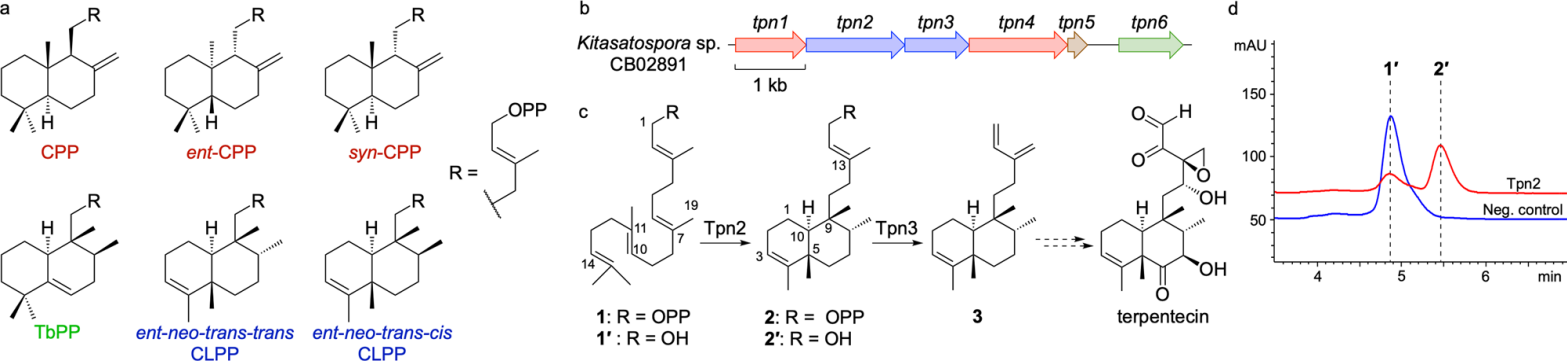
Proposed biosynthesis of terpentecin. **a** Structures of labdane (red), halimane (green), and clerodane (blue) skeletons. **b** The *tpn* BGC consists of two TSs (blue), two P450s (red), a ferredoxin (brown) and a GGPP synthase (green); mevalonate pathway genes responsible for production of the terpene precursors are found downstream. **c** Tpn2 first cyclizes GGPP into terpentedienyl diphosphate (**2**), a clerodane diterpene. Tpn3 eliminates the diphosphate of **2** to produce terpentetriene (**3**). The oxidation of **3** to terpentecin is still unknown. Carbon numbering for both **1** and **2** are shown. **d** HPLC analysis of the Tpn2 in vitro reaction confirms its type II TS activity. Negative control is reaction with boiled Tpn2. Products are shown as the dephosphorylated analogs.

Due to the limited number of known bacterial terpenoids, there is a lack of general sequence-structure-function knowledge regarding the biosynthetic machinery for bacterial TSs. Only a handful of bacterial type II TSs have been functionally characterized and only three diterpene skeletons, labdane, clerodane, and halimane, are currently known to be produced by these enzymes (Fig. 1a and Supplementary Fig. 1). Enzymes that form labdane skeletons include the *ent*-copalyl diphosphate (CPP) synthase PtmT2 from platensimycin and platencin biosynthesis in *Streptomyces platensis*^16,17^, as well as other *ent*-CPP^18–20^ and *n*-CPP^21,22^ synthases. The clerodane synthase Cyc1, found in the biosynthetic gene cluster (BGC) of the antitumor antibiotic terpentecin, is proposed to bicyclize GGPP into *syn*-CPP^+^ before a cationic cascade that ends with deprotonation at C3 to form terpentedienyl diphosphate (TPP, **2**), or *ent*-*neo*-*trans*-*trans*-clerodienyl diphosphate (CLPP, Supplementary Fig. 1)^23^. Haur_2145 forms (+)-kolavenyl diphosphate, the *ent*-*neo*-*trans-cis*-CLPP diastereomer from the *n*-CPP^+^ intermediate^24^. Rv3377c, from *Mycobacterium tuberculosis*, also forms *n*-CPP^+^ but quenches the cationic cascade by deprotonation at C6 on a halimane skeleton to yield tuberculosinyl diphosphate (TbPP)^25^. Only two of these enzymes, PtmT2 and Rv3377c, have been structurally characterized^16,26^.

In this study, we aimed to understand what controls labdane, halimane, or clerodane skeletal formation in bacterial TSs. To achieve this, we structurally and biochemically investigated Tpn2, a homolog of Cyc1. We experimentally verified Tpn2 as a TPP synthase and determined its structure by X-ray crystallography at a resolution of 2.57 Å. This is only the third structurally determined bacterial type II TS and the first from any organism that produces a clerodane skeleton. Using the structural data, we performed a series of mutations to probe its mechanism and identified a single-residue switch, G485D, that controls the selectivity between clerodane and labdane formation. Finally, we used bioinformatics to categorize four major families of type II TSs in bacteria, predict labdane- vs clerodane-forming TSs in actinobacteria, and support that uncharacterized BGCs harbor novel terpenoids. This study illustrates the need for additional characterization of bacterial TSs to expand the current scope of terpene enzymology and improve functional prediction of TSs, ultimately providing opportunities for genome mining and discovery of otherwise cryptic novel terpenoids.

## Results and discussion

### Tpn2 is a terpentedienyl diphosphate synthase

We identified a BGC (*tpn*) in *Kitasatospora* sp. CB02891 identical in organization to the one reported in *Kitasatospora griseola* MF730-N6, the original terpentecin producer^5,23,27^. The *tpn* BGC includes a GGPP synthase, two TSs, two cytochrome P450 enzymes, and a ferredoxin (Fig. 1b). Both TSs, the type II TS Tpn2 and the type I TS Tpn3, are homologous to Cyc1 and Cyc2 from *K. griseola*^23,28^ with 96 and 97% sequence identities, respectively, and are proposed to form terpentetriene (**3**) from GGPP via TPP (**2**) (Fig. 1c). To confirm that Tpn2 is a TPP (**2**) synthase, we cloned and expressed *tpn2* in *E. coli* for protein production and purification (Supplementary Fig. 2). Purified Tpn2 was incubated with GGPP and HPLC and GC-MS analysis of the enzyme reaction revealed one major product with an MS fragmentation pattern supporting a clerodane diterpene (Supplementary Fig. 3). To simplify detection, reaction products were dephosphorylated and detected as their alcohol derivatives **1′** (geranylgeraniol, GGOH) and **2′** (*syn*-kolavenol) (Fig. 1d). To unambiguously confirm the function of Tpn2, **2** was isolated from large scale in vitro reactions and its structure was confirmed by ^1^H and ^31^P NMR analysis (Supplementary Figs. S4 and S5 and Supplementary Table 4).

### First crystal structure of a clerodienyl diphosphate synthase

**Fig. 2:**
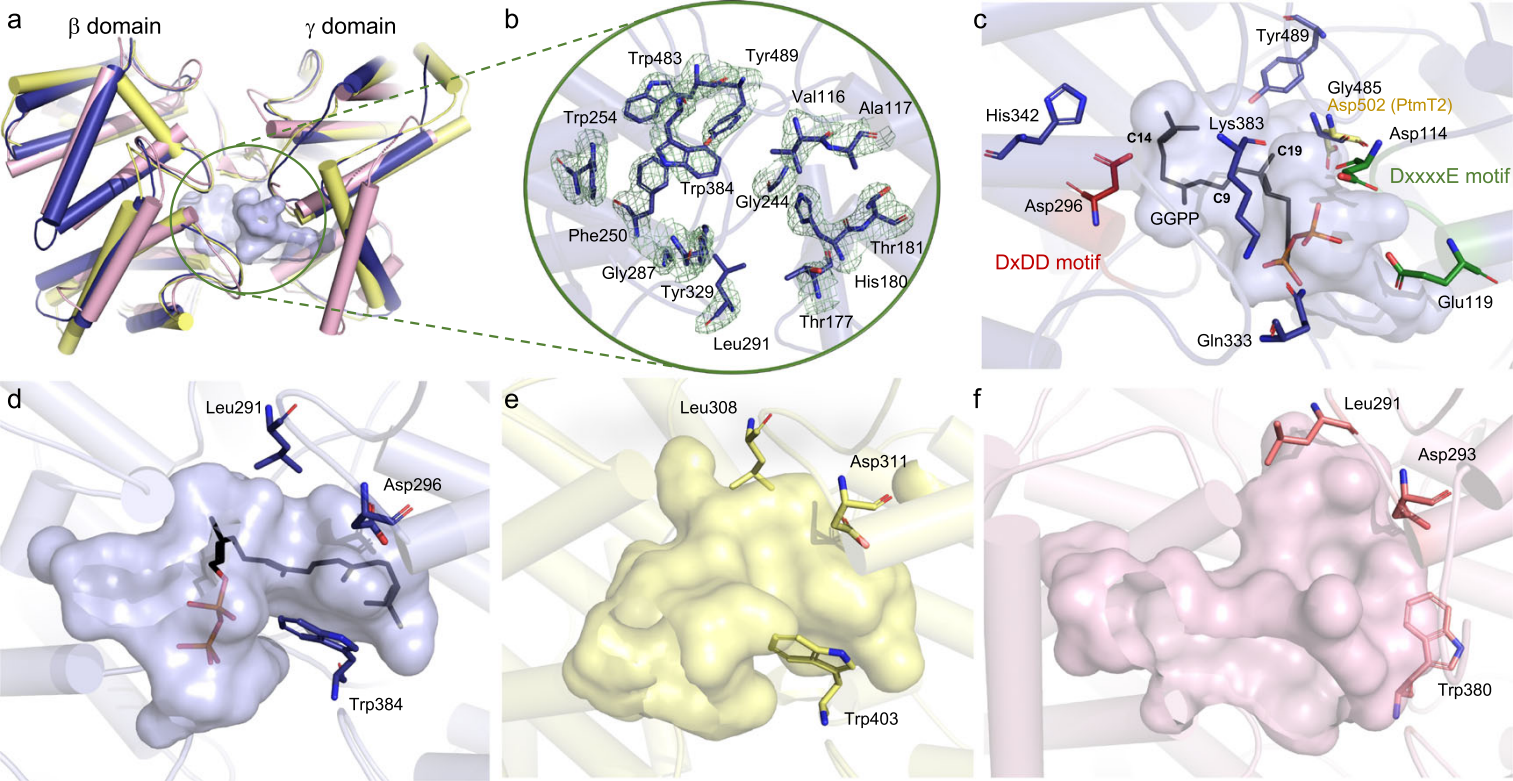
Structure of Tpn2. **a** Overall βγ didomain structure of Tpn2 (PDB ID 7XKX, blue) aligned with PtmT2 (PDB ID 5BP8, yellow) and Rv3377c (PDB ID 6VPT, pink). The active site pocket of Tpn2 is circled in green. **b** Hydrophobic residues found in the active site pocket of Tpn2. The σA-weighted difference (*mF*_*o*_ *− DF*_*c*_) omit map for Tpn2 with a 3σ contour is shown in green mesh. **c** Proposed binding mode of GGPP in the active site of Tpn2. C14 of GGPP lies 3.2 Å away from the carboxylate side chain of D296. G485, and the corresponding D502 in PtmT2, lie 3.6 Å from C19 of GGPP. Comparisons of the active site pockets of Tpn2 (**d**), PtmT2 (**e**), and Rv3377c (**f**).

We determined the crystal structure of Tpn2 at a resolution of 2.57 Å (Fig. 2 and Supplementary Table 6). As seen in PtmT2 and Rv3377c^16,26^, Tpn2 adopts the characteristic double α-barrel βγ fold with the active site residing in the interfacial cavity. As is typical for TSs from both eukaryotes and prokaryotes^8^, the active site cavity of Tpn2 is lined with both aromatic (H180, F250, W254, Y329, W384, W483, and Y489) and aliphatic (V116, A117, T177, T181, G244, G287, L291, and G485) residues to form a hydrophobic pocket suitable for binding the long nonpolar tail of the substrate and preventing water or other nucleophiles from prematurely quenching the reaction (Fig. 2b). At the bottom of the active site pocket lies the catalytically essential Asp-rich motif D^294^xDD. Docking experiments support that the central Asp, D296, is properly positioned (3.2 Å from C14) for both the protonation of GGPP (Fig. 2c) and the deprotonation of the *syn*-cleroda-13*E*-en-4-yl^+^ intermediate (TPP^+^, Supplementary Fig. 1). D296 is also suitably positioned (2.8 Å) near H342 for activation. Tpn2 also includes other conserved residues found in type II TSs including K383, which was previously proposed to interact with the negatively charged diphosphate group on the substrate in both plant and bacterial *ent*-CPPSs^16,29^.

One structural feature of type II diterpene synthases that remains ambiguous is the location of metal ions in type II TSs. Previous studies support that type II TSs are dependent on the presence of divalent ions with their absence either completely inactivating or severely diminishing activity in both plant and bacterial TSs^16,30,31^. However, because divalent metal ions are not directly involved in catalysis, it is proposed that they instead play an important role in binding the negatively charged diphosphate group of the substrate^29^. Tpn2, PtmT2, and Rv3377c each contain a D^114^xxxxE motif that resides near the opening of the active site and is proposed to be responsible for metal-binding in bacterial type II TSs (Supplementary Fig. 6)^16,26^ This is supported by molecular modeling in both Tpn2 (Fig. 2c) and Rv3377c^26^, as well as site-directed mutagenesis in PtmT2^16^. As with PtmT2 and Rv3377c, no bound divalent metal ions can be inferred in the electron density of the crystal structure of Tpn2, leaving the exact binding mechanism of divalent ions in bacterial type II TSs undetermined.

Overall, Tpn2 is structurally homologous to both PtmT2 and Rv3377c with root-mean-square deviations (rmsd) of 1.25 and 1.89 Å, respectively, for Cα atoms (Fig. 2a). Tpn2 shares 33% sequence identity and 45% similarity with PtmT2 and 26% identity and 38% similarity with Rv3377c (Supplementary Fig. 6). A few key differences between these three enzymes give rise to changes in product selectivity. Analysis of the active site cavity of Tpn2 revealed that Tpn2 and PtmT2 possess cavities of similar size and shape; however, its pocket is much narrower than the one seen in Rv3377c (Fig. 2c–e). Most notably, W384 and L291 in Tpn2 (W380 and L290 in Rv3377c) protrude into the domain interface resulting in a narrower cavity that likely restricts the conformation of GGPP. This establishes a template for *syn*-CPP^+^ formation, positioning residues that may facilitate deprotonation near the proposed location of the cationic intermediates. Previous structural, docking, and mutation studies support that Y479 is responsible for deprotonation of C6 of the halima-13-en-5-yl^+^ intermediate (TbPP^+^) to form TbPP (Supplementary Fig. 1)^26,32^. While this residue is strictly conserved across type II TSs in bacteria, its function is not. Despite the presence of an analogous Tyr residue in Tpn2 (Y489), no detectable halimadienyl diphosphate is formed in the Tpn2 reaction. Structurally, this is supported by the docking pose of GGPP and a distance of 6.2 Å between Y489 and C9 of GGPP (C6 of the halimane scaffold); this distance is only 2.7 Å in Rv3377c (Fig. 2c)^26^.

**Fig. 3:**
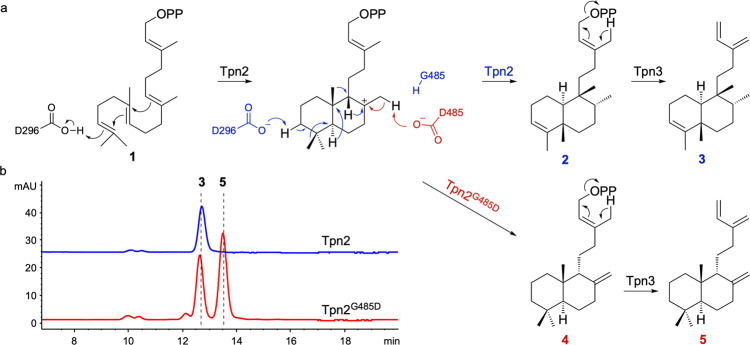
G485 is a single residue switch controlling labdane and clerodane formation in Tpn2. **a** Cyclization mechanism of Tpn2 (blue) and Tpn2^G485D^ (red). **b** HPLC analysis of Tpn2-Tpn3 and Tpn2^G485D^-Tpn3 fusion proteins in the *E. coli* MKI4 system.

With no other basic residues appropriately positioned for deprotonation, Tpn2 forces *syn*-CPP^+^ through a series of methyl and hydride shifts to access the recently deprotonated D296 (Fig. 3). This mechanism, where the central Asp acts as both initiating acid and quenching base, is consistent with labeling studies of type II mutant TSs that produce CLPPs^33,34^. Given the high conservation in active site cavity shape and residues between Tpn2 and PtmT2, we reasoned that the residue at 485 controls product determination. In PtmT2 and *At*CPS, deprotonation of the CPP^+^ at C17 is proposed to occur by a water molecule that is hydrogen bonded to D502^16,29^. Lacking an analogous Asp on the side opposing the DxDD motif, Tpn2 instead has a G485 situated 3.6 Å from C19 of the substrate (Fig. 2c). Given the similarities in active site cavity and residues, and the position of G485 in relation to C7 of GGPP (C8 of the labdane scaffold), we hypothesized that a basic residue at 485 would result in deprotonation of C19 and formation of CPP.

### Structure-guided mutagenesis converts Tpn2 into a *syn*-copalyl diphosphate synthase

To assess whether we could stimulate labdane production by Tpn2, we created a G485D mutant. Preliminary in vitro reactions supported that the G485D mutant produced at least two new products compared to native Tpn2 (Supplementary Fig. 7). Due to its low activity, we were unable to isolate enough product for structure determination. To overcome this challenge, we employed a terpenoid overproduction system in *E. coli*. First, while it has been previously shown that endogenous *E. coli* phosphatases can remove the diphosphate moiety^25,35^, we envisaged that using Tpn3 to eliminate the diphosphate group via its native type I TS activity may provide a more efficient route for the production and isolation of diterpenes. In addition, it was reported that the Tpn3 homolog Cyc2 accepts both labdane and clerodane scaffolds^36^, demonstrating the potential substrate promiscuity needed for the investigation of Tpn2 mutants. Second, we utilized our recently reported MKI4 system, a GGPP overproduction system that leverages two promiscuous kinases to phosphorylate exogenously added isoprenol^37,38^, to further enhance diterpene production in vivo (Supplementary Fig. 8).

We built a series of plasmids with *tpn2* and *tpn3*, including a genetically fused *tpn2*-*tpn3* construct^39^, to test for terpentetriene (**3**) production in *E. coli* (Supplementary Table 3). Bacterial TSs are excellent candidates for building multidomain enzymes as fusing a type II and type I TS mimics the structure of naturally occurring bifunctional TSs from plants and fungi^8,40,41^. Our initial screen found that the *tpn2-tpn3* fusion led to the highest production of **3**, likely due to the increased proximity of the TS active sites (Supplementary Fig. 9). As expected, the major product was **3**, isolated at a yield of 9.0 mg L^−1^ and structurally confirmed via ^1^H and ^13^C NMR (Supplementary Table 4, Supplementary Figs. 3, 10, and 11). This confirmed the function of Tpn3 as a terpentetriene synthase. Two additional products, **6** and **7**, were also isolated with GC-MS and NMR confirming them as α- and β-springene, respectively (Supplementary Figs. 12–14). Consistent with our results, it was previously shown that Cyc2 eliminates the diphosphate moiety from GGPP, resulting in the formation of various acyclic diterpenes^36^.

With an efficient diterpene production strategy in place, we cloned the Tpn2^G485D^-Tpn3 fusion construct and expressed it in the MKI4 system. GC-MS analysis of the crude extract showed the production of three new products in addition to **3**, **6**, and **7** (Supplementary Fig. 12). All three new products showed fragmentation patterns representative of labdane diterpenes (Supplementary Fig. 15). The major product, **5**, exhibited an *m/z* of 272.15; the two minor products exhibited *m/*z values of 290.39 and 272.28, respectively. NMR and optical rotation analysis of **5**, which was isolated at a yield of 8.0 mg L^−1^, revealed it to be *syn*-sclarene (Fig. 3, Supplementary Figs. 15–20, and Supplementary Table 5, also referred to as griseolaene)^36,42^. The formation of *syn*-sclarene by Tpn2^G485D^-Tpn3 indicates that Tpn2^G485D^ preferentially forms *syn*-CPP (**4**). Thus, G485D acts as a single-residue switch, exhibiting sole control over the formation of the clerodane TPP or labdane *syn*-CPP (Fig. 3). We also probed if mutation to a Glu residue would affect the formation of **5**. Tpn2^G485E^-Tpn3 also produced both **3** and **5**, but at a ratio of 2.3:1 (Supplementary Fig. 21); Tpn2^G485D^-Tpn3 gave a ratio of 1.6:1. It is interesting that Tpn2^G485D^ and Tpn2^G485E^ are still able to produce **2**, albeit not surprising given that they retain both the active site contour and D296. Previous quantum chemical calculations also suggested that there is not a large energetic barrier between the initial bicyclization of GGPP into *syn*-CPP^+^ and the final methyl shift to form the *syn*-cleroda-13*E*-en-4-yl^+^ final intermediate^43^. Conversely, although Tpn2 has all the machinery necessary to produce the halimane diterpene TbPP, i.e., D296 for initiation, a non-basic residue at G485 (the analogous residue in Rv3377c is A475), and the proposed base Y489 (Y479 in Rv3377c), Tpn2 does not produce any halimane compounds. In addition, the Tpn2^Y489A^ mutant is completely inactive (Supplementary Fig. 7). This supports that Y489 is not involved as a general base in the Tpn2 reaction, but instead may play an important role in forming the active site contour. Therefore, it is evident that the structural differences between Tpn2 and Rv3377c lead to significant differences in substrate binding and therefore control product specificity in these two TSs.

### Site-directed mutagenesis supports the mechanism of clerodane biosynthesis in bacteria

While the residue analogous to H180 in Tpn2 is conserved among labdane synthases from plants, clerodane synthases in plants generally include a Phe or Tyr residue in this position^44,45^. Exchanging this His in some plant TSs switched product selectivity from CPP to a CLPP or halimane diphosphate (HPP). For example, AtCPS^H263Y^ switched activity from *ent*-CPP to (–)-kolavenyl diphosphate (*neo*-*trans-cis*-CLPP)^34^ and OsCPS4^H501F^ from *syn*-CPP to *syn*-HPP^43^. Correspondingly, SdCPS2^F255H^ from *Salvia divinorum* switched its activity from neo-*trans*-*cis*-TPP to *ent*-CPP^46^. It is therefore intriguing that Tpn2 possesses a His residue at this position despite being a clerodane synthase. We created the Tpn2^H180F^ and Tpn2^H180V^ mutants to examine if it would affect product formation; however, these mutants were inactive (Supplementary Fig. 7). These results imply that while this residue may give insight into product selectivity in plant TSs, the same logic is not applicable to bacterial TSs.

We made additional mutations in the Tpn2 active site to probe the importance of various residues for catalysis (Supplementary Fig. 7). As expected, mutation of the central Asp, D296A, results in complete inactivation of the enzyme. It has been proposed that the central Asp is activated by water in cyclases such as SHC or by an adjacent His residue in PtmT2^16,47^. Accordingly, mutation of H342 to Ala or Phe abolished any product formation. These results, along with the structural data, support that D296 is responsible for initiation via protonation and that H342 is required to activate D296 for catalysis. Finally, mutation of the conserved K383 also resulted in inactivation of the enzyme (Supplementary Fig. 7), consistent with previous studies that suggested this residue is involved in diphosphate binding^16^.

### Bioinformatics analysis of type II TSs highlights terpenoid biosynthetic potential in bacteria

**Fig. 4:**
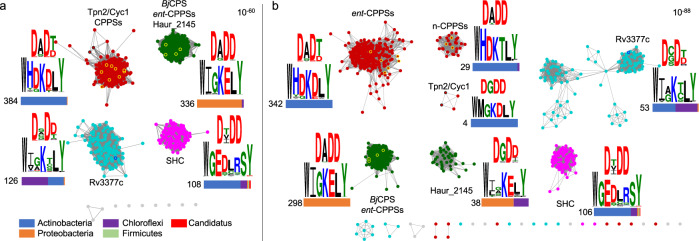
Bioinformatics of bacterial type II TSs. The 964 identified type II TSs separate into four major families in an SSN at an e-value of 10^−60^ (**a**) and six major families at an e-value of 10^−88^ (**b**). Functionally characterized TSs producing labdane (yellow), clerodane (black), or halimane (blue) are outlined. Sequence logos highlighting the conservation of the DxDD and Asp/Gly-containing motifs are shown. The number of TSs in each family and taxonomic distributions are shown below the sequence logos.

With a single residue mutation, Tpn2^G485D^ became the first *syn*-CPP synthase from bacteria. Such a simple change supports the notion that bacteria possess uncharacterized type II TSs that possess new functions. To assess this hypothesis, we performed a comprehensive bioinformatics analysis of all canonical type II TSs in bacteria. Using the Enzyme Function Initiative - Enzyme Similarity Tool^48^, a total of 964 type II TSs were identified from a BLAST search of Tpn2 given a minimum e-value threshold of 5; 876 of these were unique sequences. These TSs separated into four major subfamilies in a sequence similarity network (SSN) at an e-value of 10^−60^ (Fig. 4a). Tpn2 clustered with actinobacterial CPP synthases (384 members), gibberellin-associated *ent*-CPP synthases clustered with Haur_2145 (336 members), Rv3377c was found in its own subfamily consisting of terrabacterial TSs (126 members), and one family, of mainly actinobacterial TSs, has no characterized enzymes (108 members) but is highly similar to squalene-hopene cyclases (SHCs) from other bacteria^49^. At an e-value of 10^−88^, Tpn2, the *n-*CPP synthases, and Haur_2145 were each found within distinct clusters (Fig. 4b).

Sequence analysis of these four major subfamilies revealed both conservation and variation of key motifs and residues (Fig. 4). As expected for type II TSs, the central Asp is almost 100% conserved in all families. In the Tpn2/CPP synthase family, H180 (95.6%) and Y489 (96.9%) are all highly conserved; this is consistent with mutations at these positions rendering Tpn2 inactive. The residue at position 485 is either Asp (84.1%) or Gly (13.3%) implicating that most of these TSs form labdanes. In the Rv3377c family, the basic Y479 (92.1%) is conserved; A475, the structurally analogous position to G485 in Tpn2, shows more variation with Gly (58.7%), Ala (25.4%), Asp (6.3%), and Ser (3.2%). Overall, this is consistent with most members of this family forming a halimane skeleton. Contradictory to the actinobacterial CPP synthases, the cluster containing the gibberellin-associated *ent*-CPP synthases and Haur_2145 mostly possess Gly (91.1%) at the position analogous to G485. While this makes sense for the clerodane-forming Haur_2145, this suggests that the active site pocket of the gibberellin-associated TSs may be altered from the actinobacterial orthologues. Finally, the putative SHCs have significantly different sequences (≅30% identity with ≅30% coverage) than the characterized TSs although they have strictly conserved (>99%) Glu and Tyr in a similar C-terminal motif.

To assess the diversity of BGCs containing the 964 type II TSs, we constructed a genome neighborhood network (GNN) from the SSN at an e-value of 10^−88^ (Supplementary Fig. 22)^48^. The subfamily with the most genetic diversity is the actinobacterial *ent*-CPPS cluster. Of the 342 BGCs, 67% encode a type I TS and 14% encode a likely UbiA cyclase; this implies that the remaining 67 BGCs do not use a second cyclase, unless a noncanonical TS is utilized^14^. Many (44%) of the BGCs that contain homologs of Rv3377c have equivalents of Rv3378c, the adenosine prenylation enzyme^50^, although 33% of these contain type I TSs. The family containing Haur_2145 is similar with 21% containing Rv3378c homologs and 36% containing type I TSs. The BGCs containing Tpn2/Cyc1, *n*-CPP synthases, gibberellin-associated *ent*-CPP synthases, and SHCs are all extremely conserved in gene presence and organization supporting production of tepentecin, cyslabdan/labdanmycins, gibberellins and hopanoids, respectively^23,51–54^. These findings suggest that there is more biosynthetic diversity than what is currently known for bacterial diterpenoids, e.g., *ent*-CPP may not always be transformed into the polycyclic kauranes, atisanes, and pimaranes of currently known bacterial diterpenoids. Add this to the recent discoveries of a monodomain type II TS from cyanobacteria^55^ and a bifunctional sesqui-TS that belongs to the haloacid dehalogenase-like hydrolase superfamily^56^ and the terpenoid biosynthetic potential of bacteria is likely severely underestimated.

In summary, we determined the structure of the TPP synthase Tpn2, a clerodane-forming TS, and mapped out residues that are essential for catalysis and those that control the selectivity of product formation. We identified a single-residue switch, G485D, that converted a clerodane-specific TS into a labdane-selective TS. This residue is different from those previously seen in plant TSs that controlled clerodane vs labdane formation, highlighting the sequence-function differences between eukaryotic and prokaryotic TSs and illustrating the need to continue characterizing known and new bacterial TSs. Notably, Tpn^G485D^ is the first *syn-*CPP synthase from bacteria. This strongly implies that the functional scope of bacterial TSs is much broader than currently known and supports continued discovery of novel TSs and terpenoids in bacteria.

## Methods

### General experimental procedures

^1^H and ^13^C NMR experiments were performed in D_2_O or CDCl_3_ at 600 MHz for ^1^H and 151 MHz for ^13^C nuclei on a Bruker Avance III Ultrashield 600. 2D NOE experiments were performed on a Bruker Avance-III-800 console equipped with a Bruker 18.8 T/54 mm Ascend Magnet at 25 °C. Preparative HPLC was conducted using an Agilent 1260 Infinity II LC equipped with an Agilent Eclipse XDB-C18 column (250 mm × 21.2 mm, 7 μm). Analytical HPLC was performed on an Agilent 1260 Infinity II system equipped with an Agilent 5 HC-C18(2) column (150 × 4.6 mm, 5 μm). GC-MS experiments were run on a ThermoScientific Trace GC Ultra spectrometer equipped with an Rxi-5MS column (Restek Corp., 30 m × 0.25 mm i.d., 0.25 μm df). The injection temperature was 250 °C, electron ionization was performed with 70 eV, ion source temperature was 250 °C, transfer line temperature was 280 °C, and mass scan range was from m/z 30–500 at 1500 μ s^−1^. The program held at 50 °C for 3 min, increased the temperature at a rate of 20 °C min^−1^ up to 300 °C, and then was maintained at 300 °C for 3 min. Optical rotations were measured on a JASCO P-1010 polarimeter.

### Gene cloning

Strains and plasmids used in this study are listed in Supplementary Tables 1 and 2, respectively. The *tpn2* gene from *Kitasatospora* sp. CB02891 (UniProt accession A0A2M9LDX2, cloned starting at Met9) was amplified by PCR from genomic DNA using Q5 DNA polymerase (NEB) with primers pRSFtpn2_F and pRSFtpn2_R (Supplementary Table 3) following the manufacturer’s protocols. The PCR product was purified and treated with T4 polymerase for cloning into pBS3080 (ref. ^57^) following ligation independent procedures^58^ to create pJR2001. For site-directed mutagenesis, the *tpn2* gene was amplified in two steps by primer extension^59^ using primers pRSFtpn2_F and pRSFtpn2_R, with internal primers containing the desired mutation(s) as listed in Supplementary Table 3. The mutant genes were then cloned into pBS3080 as described previously to yield pJR2002–pJR2012. The *tpn2* G485E mutant was amplified by primer extension using primers Tpn2-3_F and Tpn2_HindIII_R and internal primers containing the mutation. The gene was then cloned into pCDF-Duet at the *Asc*I and *Hind*III sites using T4 DNA Ligase to afford pJR2019.

For in vivo production, *tpn2* (Tpn2 = A0A2M9LDX2), *tpn3* (Tpn3 = A0A2M9LE16), and *tpn6* (Tpn6 = A0A2M9LDW8) were amplified from *Kitasatospora* sp. CB02891 genomic DNA using Q5 DNA polymerase and primers Tpn2-3_F and Tpn1-3_R for *tpn2* and *tpn3* and primers Tpn6_F and Tpn6_R for *tpn6*. *tpn2/tpn3* and *tpn6* were inserted into separate multiple cloning sites in pET-Duet via restriction enzyme digestion using *Asc*I and *Hind*III for the former and *Nde*I and *Pac*I for the latter, followed by ligation via T4 DNA Ligase. For the addition of a second ribosome-binding site and fusion protein construction, *tpn2* was amplified from pJR2001 using Q5 DNA polymerase and primers NcoI_ala_Tpn2_F and either Tpn2_RBSOP_R or Tpn2_GSlink_R, respectively. The full length *tpn3* gene was similarly amplified using either primer RBSOP_Tpn3_F or GSlink_Tpn3_F and Tpn3_Hind_R for addition of a second ribosome binding site or flexible linker upstream of Tpn3. Each construct was amplified by primer extension with primers NcoI_ala_Tpn2_F and Tpn3_Hind_R and inserted into pCDF-Duet linearized with *Nco*I using T5 exonuclease-dependent assembly (TEDA)^60^, affording pJR2015. For antibiotic selection compatibility, wild-type (WT) *tpn2* and the G485D mutant were subcloned by digestion of pJR2001 and pJR2011 with *Bam*HI and *Pac*I and ligated into pCDF-Duet to form pJR2014 and pJR2015, respectively. For construction of the *tpn2* G485D and G485E mutant fusion proteins, mutant *tpn2* was amplified from pJR2011 and pJR2019, respectively, using primers Tpn2-3_F and Tpn2_GSlink_R. The full length *tpn3* gene was amplified from pJR2015 using primers GSlink_Tpn3_F and Tpn1-3_R. The *tpn2* mutants and *tpn3* were amplified together by primer extension using primers Tpn2-3_F and Tpn1-3_R and similarly cloned into the *Asc*I and *Hind*III sites affording pJR2018 and pJR2020.

### Gene expression and protein production

For in vitro activity assays, pJR2001 was transformed into *E. coli* NiCo cells (NEB) and grown in 500 mL of lysogeny broth (LB) at 37 °C with shaking at 200 rpm until an OD_600_ of 0.6 was reached. The culture was cooled on ice before gene expression was induced by addition of 0.5 mM isopropyl β-d-1-thiogalactopyranoside (IPTG). Flasks were grown overnight at 16 °C before the cells were harvested by centrifugation at 5000 × *g* for 20 min at 4 °C. The cell pellet was prepared for purification by resuspending in lysis buffer (50 mM Tris, 300 mM NaCl, and 10 mM imidazole, pH 8.0) and 1 mg mL^−1^ lysozyme before incubating on ice for 30 min. Cells were then sonicated and centrifuged at 10,000 × *g* for 30 min to separate soluble proteins before filtering with a 0.8 µM filter. The lysate was purified by nickel affinity chromatography and size exclusion chromatography using an AKTA FPLC with a 5 mL HisTrap column and Superdex HiScale 16/40 80 mL column (GE Healthcare), respectively. Purified Tpn2 was concentrated using an Amicon Ultra-15 concentrator (Millipore) in SEC buffer (50 mM Tris, pH 8.0) before storing aliquots at −80 °C. Protein concentration was calculated at 280 nm using a molar absorptivity constant of 98,320 M^−1^ cm^−1^. Each of the Tpn2 site-directed mutants was produced and purified as described above using nickel affinity chromatography.

### Crystallization, data collection, and structure determination of Tpn2

Purified Tpn2 was concentrated to 30.0 mg mL^−1^ in 20 mM Tris buffer (pH 8.0) with 100 mM NaCl and crystallized using the hanging drop vapor-diffusion method in a screen condition: 10 mM zinc sulfate heptahydrate, 25% v/v polyethylene glycol monomethyl ether 550, and 100 mM MES monohydrate, pH 6.5. The Tpn2 crystals were observed after two weeks. The crystals were transferred to cryoprotectant solution containing 20% glycerol prior to the X-ray data collection. The diffraction data of Tpn2 were collected at National Synchrotron Radiation Research Center (Taiwan) on the 15A1 beamline using a wavelength of 0.9732 Å with the ADSC QUANTUM 315r CCD detector. Diffraction data were indexed and scaled with HKL2000^61^. The structure of Tpn2 was solved by the molecular replacement using the structure of PtmT2 (PDB entry 5BP8) as a search model^16^. The model was refined with REFMAC^62^. The atomic coordinates and structure factors of Tpn2 were deposited in the Protein Data Bank (PDB) with the accession code 7XKX (Supplementary Data 1). Data processing and refinement statistics are summarized in Supplementary Table 6.

### Enzymatic activity of Tpn2

GGPP was synthesized as previously described in the literature^63,64^. In vitro reactions were performed in 50 mM Tris pH 6.8, containing 1 mM MgCl_2_, 5 mM β-mercaptoethanol (BME), 10% glycerol, 4 mM GGPP, and 20 µM Tpn2 in a total volume of 50 µL. Reactions were incubated for 16 h at 30 °C before undergoing dephosphorylation of reaction products via addition of Quick-CIP (NEB) and incubation following the manufacturer’s protocols. The reaction mixture was then gently extracted with 100 µL acetonitrile and 50 µL saturated NaCl before centrifugation for 1 min. The organic layer was then removed and injected on an HPLC system using an isocratic method of 95% acetonitrile in H_2_O. The substrate and product were detected at 210 nm as the dephosphorylated analogs **1′** and **2′** and eluted at 5.4 min and 6.0 min, respectively.

TPP (**2**) was isolated by scaling up in vitro reactions to yield approximately 0.5 mg of the product. Reactions were incubated for 16 h at 30 °C before extraction with equal volume of acetonitrile and placing on ice for 1 h. TPP (**2**) was purified via preparative HPLC using a solvent gradient of 5–50% acetonitrile in NH_4_HCO_3_. Fractions containing **2** were combined and dried down via rotary evaporator (Supplementary Figs. 3, 4).

### In vivo product isolation and determination

For in vivo product isolation, pJR2014–pJR2018 were individually co-transformed along with pJR1064 into *E. coli* BL21 Star (DE3) (Invitrogen) and grown in 9–12 L terrific broth (TB) at 37 °C with shaking at 200 rpm until an OD_600_ of 1.0 was reached. The cultures were cooled on ice before gene expression was induced by the addition of 0.5 mM IPTG and 5 mM isoprenol was supplemented in the culture. Flasks were shaken for 72 h at 28 °C before harvesting the cells by centrifugation. For the isolation **2′**, **3**, **5**, **6**, and **7**, the cell pellets were extracted with 1:1 methanol: acetone and the organic layer dried under air at room temperature. The extract was then redissolved in ethyl acetate and purified via silica chromatography with a hexanes mobile phase before additional purification via preparative HPLC as previously described. Fractions containing the desired products were combined and dried down on a rotary evaporator at 70 mbar at 20 °C.

Compounds **3**, **5**, **6**, and **7** were confirmed via NMR. The ^1^H and ^13^C spectra of **3** matched literature values (Supplementary Figs. 11, 12)^28^. The structure of *syn*-sclarene (**5**) was confirmed via additional NMR experiments, including ^1^H and ^13^C, which matched literature values^36^, ^1^H-^1^H COSY, HSQC, and HMBC (Supplementary Figs. 13–16, Supplementary Table 4). The absolute stereochemistry of **5** was confirmed by 2D NOESY and optical rotation experiments (Fig. 3 and Supplementary Fig. 17), the latter of which gave an ${[\alpha ]}_{{{{{{\rm{D}}}}}}}^{21}$  = +2.269 (c 0.005, CHCl_3_). Compound **2′** was confirmed via purification and comparison to in vitro reaction data via analysis by HPLC.

Yields were calculated by making a calibration curve using purified **3** and **5** and injecting on HPLC. Area under the curve and injection concentration was used to plot a standard curve. Tpn2-Tpn3 and Tpn2^G485D^-Tpn3 fusion proteins were fermented and extracted as previously described. Crude extracts were injected on HPLC and the area under the curve at λ = 210 nm was used to calculate yield.

### Computational modeling

The structure of the ligand, GGPP, was optimized for docking using the default parameters of MM2 energy minimization in ChemDraw 3D. This was saved in SDF format, then converted to pdbqt in OpenBabel^65^. The receptor was prepared for docking in AutoDockTools 1.5.6 (ref. ^66^); hydrogen atoms were added and merged and Gasteiger charges calculated before generating the final pdbqt file. The ligand and receptor were docked using AutoDock Vina^67^. Docking results were generated for a grid box of 30 × 25 × 20, default grid spacing of 0.375 Å, and exhaustiveness = 20, using the coordinates of the central Asp side chain oxygen atom as the grid box center to ensure the entire active site was encompassed within the grid box.

### Bioinformatics

The sequence alignment of plant and bacterial type II TSs obtained by aligning sequences from UniProt using ClustalW^68^ and visualized in ESPript 3.0 (ref. ^69^). Consensus sequence logos of motifs were depicted with partial aligned sequences using WebLogo 3 (ref. ^70^). The collection of bacterial type II TSs for sequence similar network (SSN) analysis was achieved using the Enzyme Function Initiative (EFI) Enzyme Similarity Tool (EST)^48^ with Tpn2 as the query sequence, taxonomy filter of “bacteria”, and an e-value of 5. The SSNs and genome neighborhood networks (GNNs), created using the SSN Cluster Hub-Nodes function, were generated using EFI-EST and visualized in Cytoscape 3.9.1 (ref. ^71^). Taxonomic distribution of each protein family was determined using the taxonomy function on EFI-EST.

### Reporting summary

Further information on research design is available in the Nature Research Reporting Summary linked to this article.

## Supplementary information

Peer Review File

Supplementary Information

Description of Additional Supplementary Files

Supplementary Data 1

Supplementary Data 2

Reporting Summary

## Data Availability

All data generated during this study are available either in the main text, supplementary materials, or are deposited in online repositories. The atomic coordinates and structure factors of Tpn2 (Supplementary Data 1) were deposited in the Protein Data Bank with the accession code 7XKX.
