# Supplementary material for: Structure-guided product determination of the bacterial type II diterpene synthase Tpn2
Source: Commun Chem. 2022 Nov 8;5:146. doi: 10.1038/s42004-022-00765-6 (PMC9814783; doi:10.1038/s42004-022-00765-6)
Supplement: Supplementary file 2 — Supplementary Information [file 42004_2022_765_MOESM2_ESM.pdf]

# Supplementary Information

# Structure-guided product determination of the bacterial type II diterpene synthase Tpn2

*Emma A. Stowell,*[a] *Michelle A. Ehrenberger,*[a] *Ya-Lin Lin,*[b] *Chin-Yuan Chang,**[b,c] *and Jeffrey D. Rudolf**[a]

[a]Department of Chemistry, University of Florida, Gainesville, Florida, 32611, USA

[b]Department of Biological Science and Technology, National Yang Ming Chiao Tung University, Hsinchu 30010, Taiwan, ROC

[c]Center for Intelligent Drug Systems and Smart Bio-devices, National Yang Ming Chiao Tung University, Hsinchu 30010, Taiwan, ROC

*Correspondence to E-mail: jrudolf@chem.ufl.edu; cycytl@nctu.edu.tw

**Table of Contents**

**Supplementary Table 1.** Strains used in this study ........ 3

**Supplementary Table 2.** Plasmids used in this study ........ 3

**Supplementary Table 3.** Primers used in this study ........ 4

**Supplementary Table 5.** $^{1}$H NMR (600 MHz) and $^{13}$C NMR (151 MHz) spectroscopic data for *syn*-sclarene (**5**) ........ 7

**Supplementary Table 6.** Data collection and refinement statistics for Tpn2 (molecular replacement). .... 8

**Supplementary Figure 1.** Bacterial type II TSs. ........ 9

**Supplementary Figure 2.** SDS-PAGE analysis of purified proteins ........ 10

**Supplementary Figure 3.** EI mass spectra of **2′**, **3**, and **5** ........ 11

**Supplementary Figure 4.** $^{1}$H NMR spectrum of **2** in $D_2O$ (600 MHz) ........ 12

**Supplementary Figure 5**. $^{31}$P NMR spectrum of **2** in $D_2O$ (243 MHz). ........ 13

**Supplementary Figure 6.** Sequence alignment of selected bacterial and plant type II TSs ........ 14

**Supplementary Figure 7.** HPLC chromatograms showing in vitro enzyme reactions of Tpn2 and mutants after desphosphorylation ........ 17

**Supplementary Figure 8.** Scheme of the in vivo terpenoid upregulation system ........ 18

**Supplementary Figure 9.** HPLC analysis showing production of terpentetriene (**3**) in vivo using the MKI4 system. ........ 19

**Supplementary Figure 10.** $^{1}$H NMR spectrum of terpentetriene (**3**) in $CDCl_3$ (600 MHz) ........ 20

**Supplementary Figure 11.** $^{13}$C NMR spectrum of terpentetriene (**3**) in $CDCl_3$ (151 MHz) ........ 21

**Supplementary Figure 12.** GC-MS analysis of in vivo products from Tpn2-Tpn3 and Tpn2$^{G485D}$-Tpn3 fusion proteins using the MKI4 system. ........ 22

**Supplementary Figure 13.** $^{1}$H NMR spectrum of α-springene (**6**) in $CDCl_3$ (600 MHz) ........ 23

**Supplementary Figure 14.** $^{1}$H NMR spectrum of β-springene (**7**) in $CDCl_3$ (600 MHz) ........ 24

**Supplementary Figure 15.** $^{1}$H NMR spectrum of *syn*-sclarene (**5**) in $CDCl_3$ (600 MHz) ........ 25

**Supplementary Figure 16.** $^{13}$C NMR spectrum of *syn*-sclarene (**5**) in $CDCl_3$ (151 MHz) ........ 26

**Supplementary Figure 17.** HSQC spectrum of *syn*-sclarene (**5**) in $CDCl_3$ ........ 27

**Supplementary Figure 18.** HMBC spectrum of *syn*-sclarene (**5**) in $CDCl_3$. ........ 28

**Supplementary Figure 19.** COSY spectrum of *syn*-sclarene (**5**) in $CDCl_3$. ........ 29

**Supplementary Figure 20**. 2D NOESY spectrum of *syn*-sclarene (**5**) in $CDCl_3$ with key NOE correlations shown. ........ 30

**Supplementary Figure 21.** HPLC analysis of in vivo products from Tpn2$^{G485E}$-Tpn3 ........ 31

**Supplementary Figure 22.** Genome neighborhood network of bacterial type II TSs ........ 32

**Supplementary References** ........ 33

**Supplementary Table 1.** Strains used in this study

| Strain | Description | Source (Reference) |
|---|---|---|
| *E. coli* NEB Turbo | Host for general cloning | New England Biolabs |
| *E. coli* NEB NiCo21 | Host for high-level protein production | New England Biolabs |
| *E. coli* BL21 Star (DE3) | Host for high-level protein production | Invitrogen |
| *Kitasatospora sp.* CB02891 | Putative terpentecin producer, used for isolation of genomic DNA for *tpn2* and *tpn3* amplification | Gift from Shen Lab[1] |

**Supplementary Table 2.** Plasmids used in this study

| Plasmid | Description | Source (Reference) |
|---|---|---|
| pRSF-Duet | General plasmid for cloning | Novagen |
| pCDF-Duet | General plasmid for cloning | Novagen |
| pET-Duet | General plasmid for cloning | Novagen |
| pUC19 | General plasmid for cloning | Novagen |
| pET28a | Plasmid for heterologous expression in E. coli | Novagen |
| pBS3080 | Plasmid created from pRSF-duet by cloning the TEV/LIC sites; used for cloning and protein expression | (2) |
| pJR1064 | pET28a-MKI4: pET28a harboring kinases *Ec-Thi*M and *At-IPK*, *Ec-idi*, and GGPP synthase (*bnd3*). Ribosome binding sites were inserted before each gene. | (3) |
| pJR2001 | pRSF/TEV/LIC harboring *tpn2* | This study |
| pJR2002 | pRSF/TEV/LIC harboring *tpn2* (H180F) | This study |
| pJR2003 | pRSF/TEV/LIC harboring *tpn2* (H180V) | This study |
| pJR2004 | pRSF/TEV/LIC harboring *tpn2* (D296A) | This study |
| pJR2005 | pRSF/TEV/LIC harboring *tpn2* (Y329F) | This study |
| pJR2006 | pRSF/TEV/LIC harboring *tpn2* (H342A) | This study |
| pJR2007 | pRSF/TEV/LIC harboring *tpn2* (H342F) | This study |
| pJR2008 | pRSF/TEV/LIC harboring *tpn2* (H342A-D296A) | This study |
| pJR2009 | pRSF/TEV/LIC harboring *tpn2* (H342F-D296A) | This study |
| pJR2010 | pRSF/TEV/LIC harboring *tpn2* (K383A) | This study |
| pJR2011 | pRSF/TEV/LIC harboring *tpn2* (G485D) | This study |
| pJR2012 | pRSF/TEV/LIC harboring *tpn2* (Y489A) | This study |
| pJR2013 | pET-Duet harboring *tpn2*, *tpn3*, and *tpn6* for heterologous expression in *E. coli* | This study |
| pJR2014 | pCDF duet harboring *tpn2* for heterologous expression in *E. coli* | This study |

| | | |
|---|---|---|
| pJR2015 | pCDF duet harboring *tpn2* (G485D) for heterologous expression in *E. coli* | This study |
| pJR2016 | pCDF duet harboring *tpn2* and *tpn3* with an additional RBS for heterologous expression in *E. coli* | This study |
| pJR2017 | pCDF duet harboring *tpn2* and *tpn3* linked with a glycine-serine linker for heterologous expression in *E. coli* | This study |
| pJR2018 | pCDF duet harboring *tpn2* (G485D) and *tpn3* for heterologous expression in *E. coli* | This study |
| pJR2019 | pCDF duet harboring *tpn2* (G485E) for heterologous expression in *E. coli* | This study |
| pJR2020 | pCDF duet harboring *tpn2* (G485E) and *tpn3* for heterologous expression in *E. coli* | This study |

**Supplementary Table 3.** Primers used in this study

| Name | Sequence | Purpose |
|---|---|---|
| pRSFtpn2_F | aaaacctctatttccagtcgatgagtgacgccgatcggatagc | *tpn2* or mutant amplification for protein expression in *E. coli* |
| pRSFtpn2_R | ctcgatcgcgtcgggcatcagtacctgctcacgacgg | |
| H180F_F | agacggcatggttcacactcgaggc | Tpn2 mutagenesis for H180F |
| H180F_R | gcctcgagtgtgaaccatgccgtct | |
| H180V_F | agacggcatgggtcacactcgaggc | Tpn2 mutagenesis for H180V |
| H180V_R | gcctcgagtgtgacccatgccgtct | |
| D296A_F | ccgcccgacggggccgacacggcctac | Tpn2 mutagenesis for D296A |
| D296A_R | gtaggccgtgtcggccccgtcgggcgg | |
| Y329F_F | cacttcgtttcgtttccgggggagcag | Tpn2 mutagenesis for Y329F |
| Y329F_R | ctgctcccccggaaacgaaacgaagtg | |
| H342A_F | ccgtgaacgcggccgccctcgagtac | Tpn2 mutagenesis for H342A |
| H342A_R | gtactcgagggcggccgcgttcacgg | |
| H342F_F | ccgtgaacgcgttcgccctcgagtac | Tpn2 mutagenesis for H342F |
| H342F_R | gtactcgagggcgaacgcgttcacgg | |
| K383A_F | ggctgctggtacgacgcatggaatgtctcgccg | Tpn2 mutagenesis for K383A |
| K383A_R | cggcgagacattccatgcgtcgtaccagcagcc | |
| G485D_F | ccgctgtggatggacaaggacctgtac | Tpn2 mutagenesis for G485D |
| G485D_R | gtacaggtccttgtccatccacagcgg | |
| G485E_F | ccgctgtggatggaaaaggacctgtac | Tpn2 mutagenesis for G485E |
| G485E_R | gtacaggtccttttccatccacagcgg | |
| Y489A_F | caaggacctggccacgcctttccgga | Tpn2 mutagenesis for Y489A |
| Y489A_R | tccggaaaggcgtggccaggtccttg | |

| | | |
|---|---|---|
| NcoI_ala_Tpn2_F | aataaggagatataccatggcgagtgacgccgatcggatag c | *tpn2* WT amplification for in vivo expression |
| Tpn3_Hind_R | cattatgcggccgcaagctttcagcggtagcggtctgtct | *tpn3* amplification for in vivo expression |
| Tpn2-3_F | gcaggcgcgccatgagtgacgccgatcggatagc | *tpn2* or mutant amplification for in vivo expression |
| Tpn2_HindIII_R | tagcaagctttcagtacctgctcacgacgg | |
| Tpn1-3_R | cccaagctttcagcggtagcggtctgtct | |
| Tpn2_RBSOP_R | tatacgccccccctatgccttaaataca tcagtacctgctcacgacgg | *tpn2* or mutant amplification for in vivo expression and addition of RBS sequence between *tpn2* and *tpn3* |
| RBSOP_Tpn3_F | tgtatttaaggcatagggggggcgtata atgcccgacgcgatcgagtt | |
| Tpn2_GSlink_R | agaaccgccagaaccagaaccgtacctgctcacgacggcg c | *tpn2* or mutant amplification for in vivo expression and addition of linker sequence between *tpn2* and *tpn3* |
| GSlink_Tpn3_F | ggttctggttctggcggttctatgcccgacgcgatcgagtt | |
| Tpn6_F | gcacatatgtacaccgataccgccgaaac | *tpn6* amplification for in vivo expression |
| Tpn6_R | ggttaattaatcagtggttcctgagagcgac | |
| GSlink_tpn3_teda_F | ccgcttccggaggtgctggttctggttctggcggttct | *tpn3* amplification for in vivo expression |
| Tpn3_teda_R | gactctagaggatccccgggtcagcggtagcggtctgtct | |

**Supplementary Table 4.** $^{1}H$ NMR (600 MHz) and $^{13}C$ NMR (151 MHz) spectroscopic data for TPP (**2**) and terpentetriene (**3**).[a]

| No. | **2**[b] | | **3**[c] | |
|---|---|---|---|---|
| | $\delta_H$ | | $\delta_C$ | $\delta_H$ |
| 1, $CH_2$ | 1.62 (dd, $J$ = 12.7, 6.4); 1.33 (m) | | 17.8 | 1.43 (m); 1.67 (m) |
| 2, $CH_2$ | 1.89 (m); 1.95 (m) | | 26.8 | 2.00 (m); 2.15 (m) |
| 3, CH | 5.21 (br s) | | 120.2 | 5.17 (ddd, $J$ = 4.5, 2.7, 1.3) |
| 4, qC | | | 144.5 | |
| 5, qC | | | 38.3 | |
| 6, $CH_2$ | 1.35 (m) | | 30.2 | 1.46 (m); 1.50 (m) |
| 7, $CH_2$ | 1.22 (m), 1.89 (m) | | 25.6 | 1.32 (dq, $J$ = 13.9, 3.3); 1.96 (m) |
| 8, CH | 1.47 (m) | | 35.2 | 1.69 (m) |
| 9, qC | | | 37.7 | |
| 10, CH | 1.35 (m) | | 45.1 | 1.45 (m) |
| 11, $CH_2$ | 1.08 (m), 1.47 (m) | | 38.3 | 1.20 (td, $J$ = 12.8, 4.6); 1.64 (m) |
| 12, $CH_2$ | 1.95 (m) | | 24.6 | 2.22 (m); 2.18 (m) |
| 13, qC | | | 147.8 | |
| 14, CH | 5.41 (m) | | 139.2 | 6.40 (dd, $J$ = 17.6, 10.9) |
| 15, $CH_2$ | 4.38 (t, $J$ = 6.4) | | 113.0 | 5.07 (dq, $J$ = 10.8, 1.0); 5.27 (dd, $J$ = 17.6, 1.2) |
| 16, $CH_3$ | 1.65 (d, $J$ = 1.5) | | 115.2 | 5.02 (s) |
| 17, $CH_3$ | 0.85 (d, $J$ = 6.7) | | 14.9 | 0.98 (s) |
| 18, $CH_3$ | 0.98 (s) | | 20.6 | 1.08 (s) |
| 19, $CH_3$ | 1.53 (s) | | 18.0 | 1.61 (m) |
| 20, $CH_3$ | 0.86 (s) | | 20.4 | 0.99 (br s) |

[a] δ in ppm, $J$ in Hz

[b] NMR taken in $D_2O$

[c] NMR taken in $CDCl_3$

**Supplementary Table 5.** $^{1}H$ NMR (600 MHz) and $^{13}C$ NMR (151 MHz) spectroscopic data for *syn*-sclarene (**5**).[a]

| No. | **5**[b] | |
|---|---|---|
| | $\delta_C$ | $\delta_H$ |
| 1, $CH_2$ | 36.7 | 1.08 (m); 1.58 (m) |
| 2, $CH_2$ | 19.2 | 1.46 (m); 1.65 (m) |
| 3, CH | 42.7 | 1.18 (td, *J* = 13.2, 3.6); 1.42 (m) |
| 4, qC | 33.2 | |
| 5, qC | 45.8 | 1.32 (m) |
| 6, $CH_2$ | 23.7 | 1.33 (m); 1.64 (m) |
| 7, $CH_2$ | 31.62 | 2.13 (m); 2.22 (m) |
| 8, CH | 149.3 | |
| 9, qC | 58.4 | 1.61 (m) |
| 10, CH | 38.0 | |
| 11, $CH_2$ | 25.2 | 1.53 (m); 1.75 (m) |
| 12, $CH_2$ | 30.3 | 1.95 (m); 2.16 (m) |
| 13, qC | 147.2 | |
| 14, CH | 139.0 | 6.38 (dd, *J* = 17.6, 10.8) |
| 15, $CH_2$ | 113.1 | 5.06 (dq, *J* = 10.9, 1.2);<br>5.22 (dd, *J* = 17.6, 1.1) |
| 16, $CH_3$ | 115.5 | 5.02 (s) |
| 17, $CH_3$ | 109.6 | 4.59 (dd, *J* = 2.8, 1.7);<br>4.75 (t, *J* = 2.4) |
| 18, $CH_3$ | 22.2 | 0.88 (s) |
| 19, $CH_3$ | 33.5 | 0.89 (s) |
| 20, $CH_3$ | 22.4 | 0.95 (s) |

[a] δ in ppm, *J* in Hz

[b] NMR taken in $CDCl_3$

**Supplementary Table 6.** Data collection and refinement statistics for Tpn2 (molecular replacement).

| | Tpn2 |
|---|---|
| **Data collection** | |
| Space group | $P3_1$ |
| Cell dimensions | |
| $a$, $b$, $c$ (Å) | 92.54, 92.54, 127.91 |
| α, β, γ (°) | 90.00, 90.00, 120.00 |
| Resolution (Å) | 30.00–2.57 (2.67–2.57) |
| $R_{sym}$ or $R_{merge}$ | 12.0 (60.3) |
| $I$ / σ$I$ | 15.7 (3.8) |
| Completeness (%) | 99.3 (100.0) |
| Redundancy | 4.7 (5.6) |
| | |
| **Refinement** | |
| Resolution (Å) | 30.00–2.57 |
| No. reflections | 38716 |
| $R_{work}$ / $R_{free}$ | 0.256/0.288 |
| No. atoms | |
| Protein | 7443 |
| Water | 159 |
| $B$-factors | |
| Protein | 57.8 |
| Water | 50.2 |
| R.m.s. deviations | |
| Bond lengths (Å) | 0.0047 |
| Bond angles (°) | 1.36 |

**Supplementary Fig. 1.** Bacterial type II TSs.

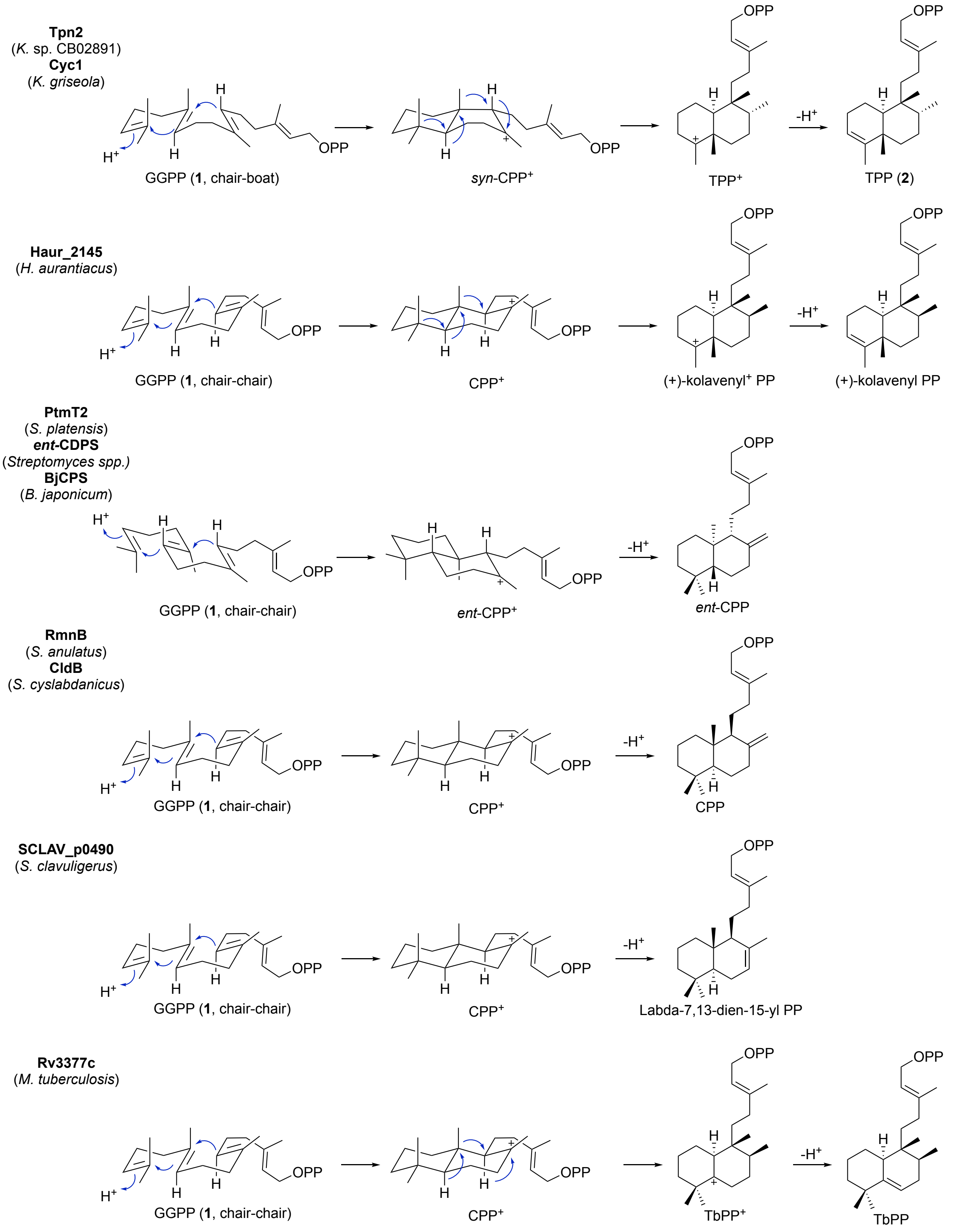

**Supplementary Fig. 2.** SDS-PAGE analysis of purified proteins. Expected size of Tpn2 is 56 kDa. Protein size analyzed using Fisher BioReagents™ EZ-Run™ Prestained Rec Protein Ladder.

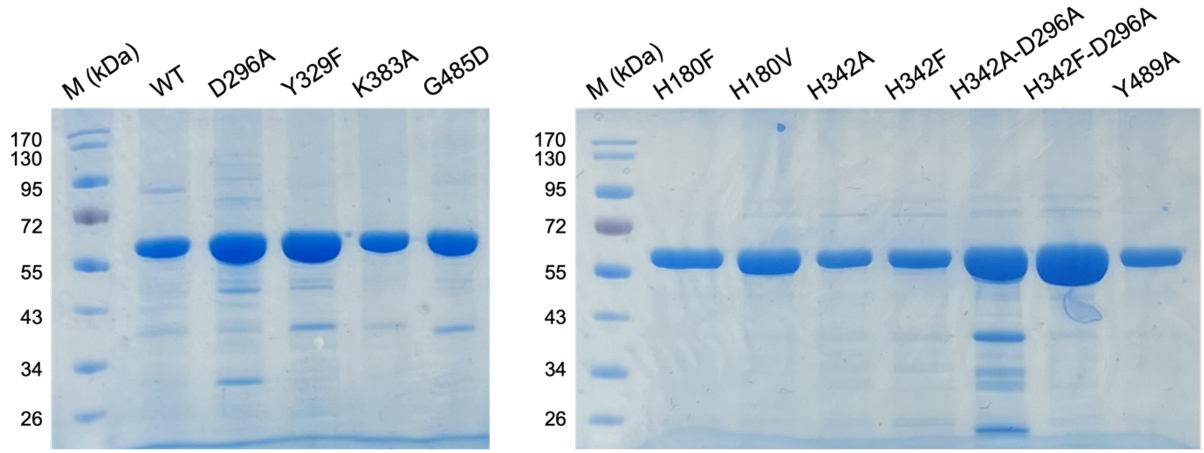

**Supplementary Fig. 3.** EI mass spectra of **2′**, **3**, and **5**. (a) EIMS **2′**: *m/z* (%): 95 (100), 107 (69), 120 (35), 41 (32), 69 (28), 189 (23), 257 (3), 290 ($M^+$, 3). (b) EIMS **3**: *m/z* (%): 107 (100), 95 (80), 120 (75), 189 (69), 41 (55), 79 (54), 67 (43), 55 (40), 132 (26), 159 (26), 257 (13), 272 ($M^+$, 3). (c) EIMS **5**: *m/z* (%): 69 (100), 41 (85), 81 (81), 93 (79), 109 (72), 257 (57), 55 (54), 123 (42), 149 (32), 272 ($M^+$, 7).

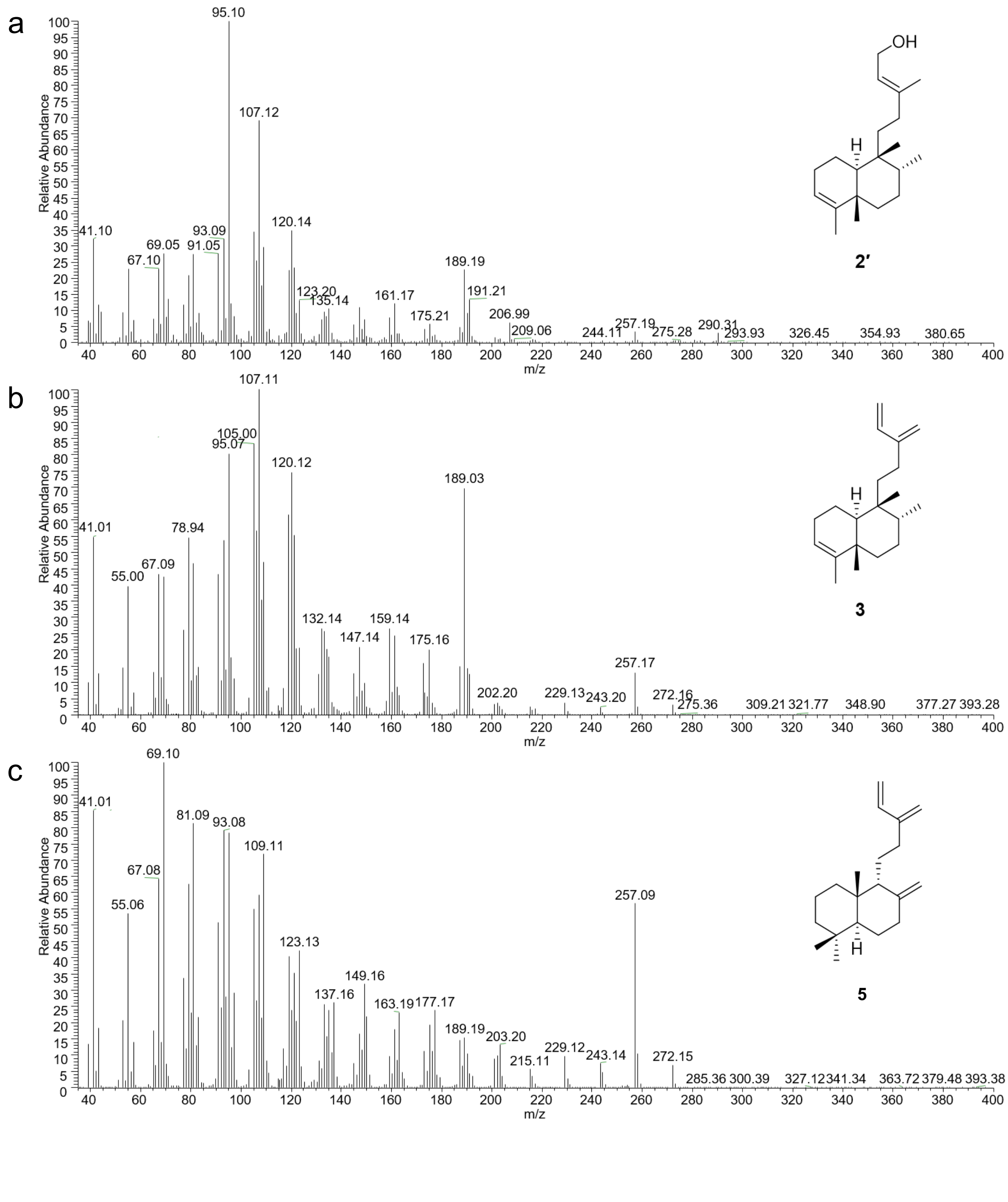

**Supplementary Fig. 4.** $^{1}H$ NMR spectrum of **2** in $D_2O$ (600 MHz). TPP (**2**) was isolated in the presence of glycerol; peaks marked with asterisks.

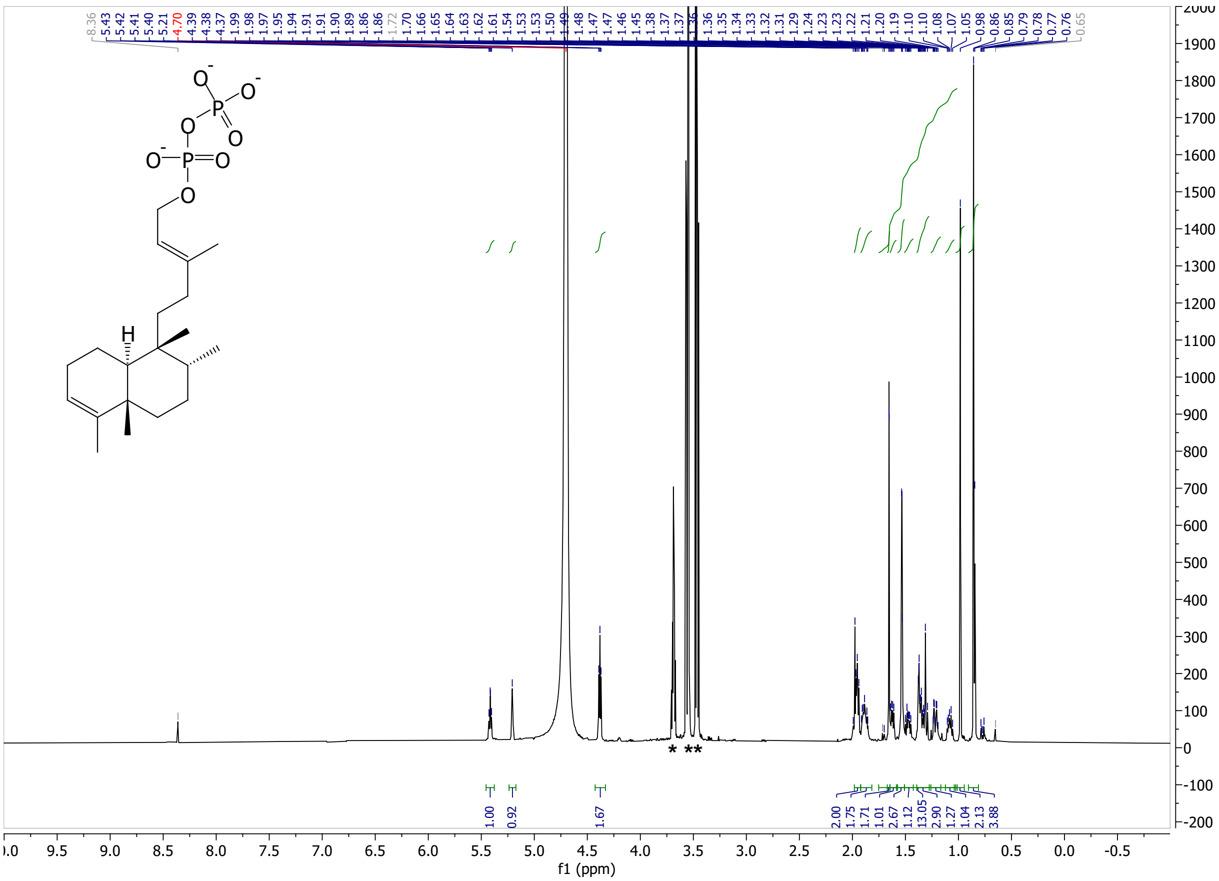

**Supplementary Fig. 5**. $^{31}$P NMR spectrum of **2** in $D_2O$ (243 MHz).

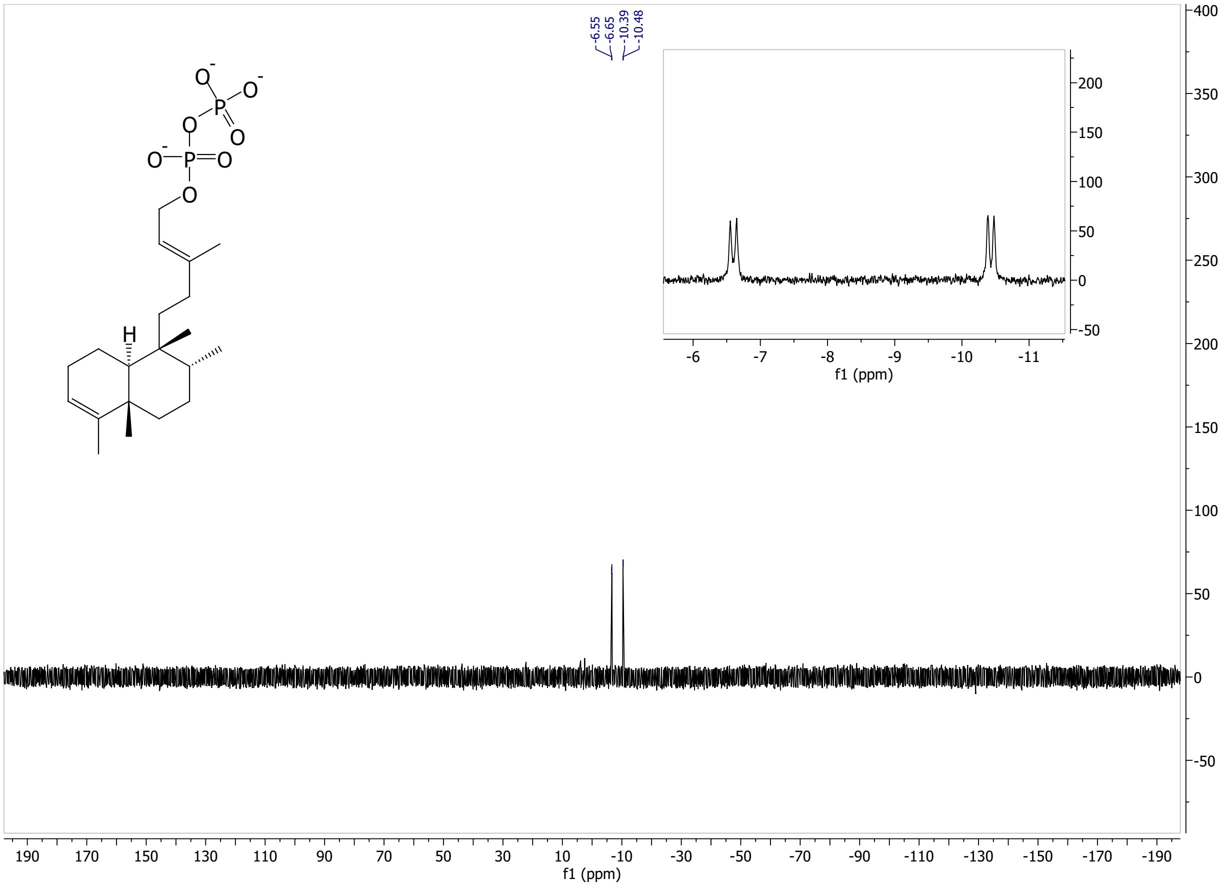

**Supplementary Fig. 6.** Sequence alignment of selected bacterial and plant type II TSs. Residues are colored based on conservation (identity: red box with white residue; similarity: red residue; similar across group: blue frame). The secondary structure of Tpn2 is shown above the alignment. The DxDD motif is in a black frame, residues mutated in this study are in green, and DxxxxE is in blue. The alignment was created with Clustal Omega[4] and rendered with ESPript.[5] The α domain of AtCPS was removed.

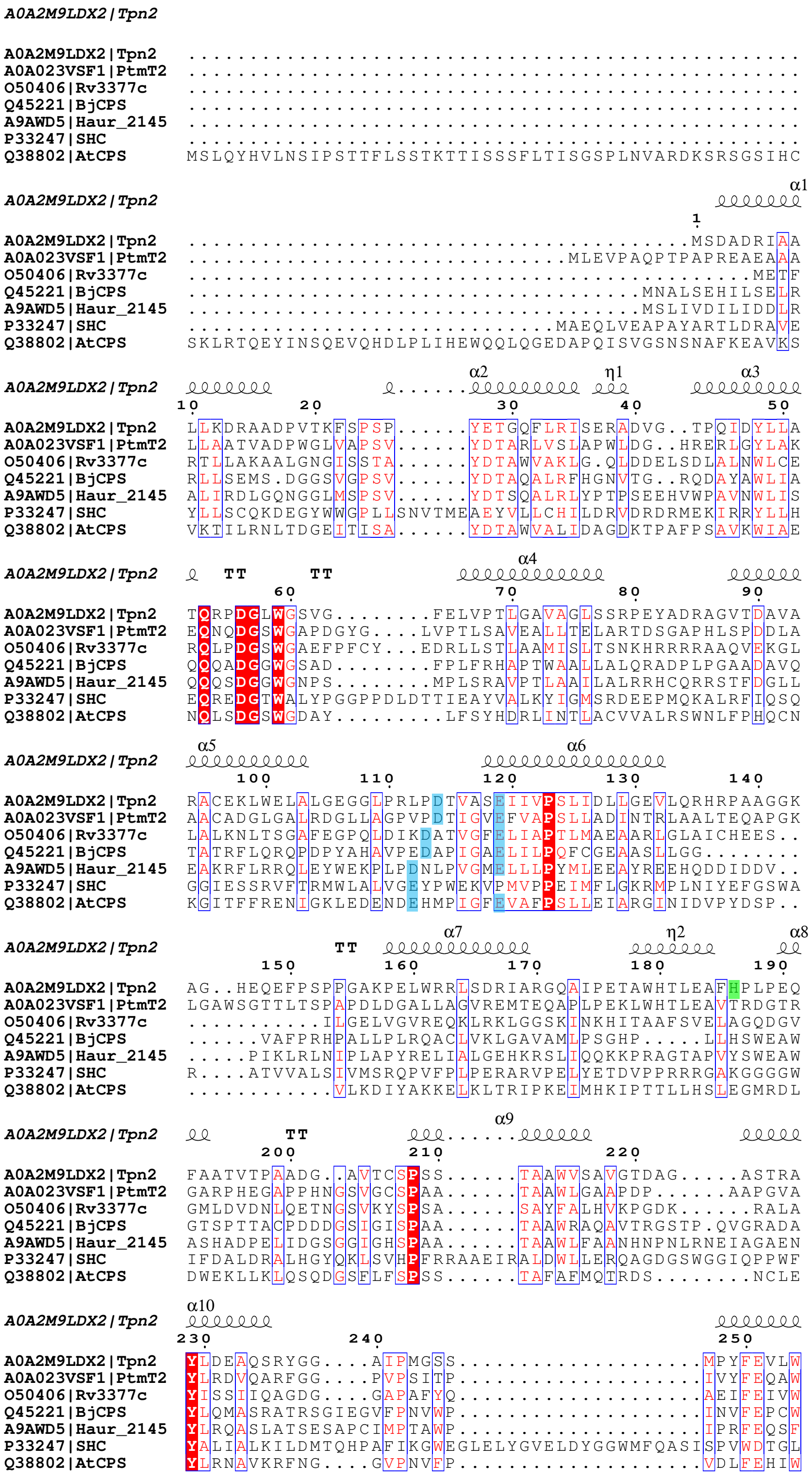

**Supplementary Fig. 6 cont.** Sequence alignment of selected bacterial and plant type II TSs.

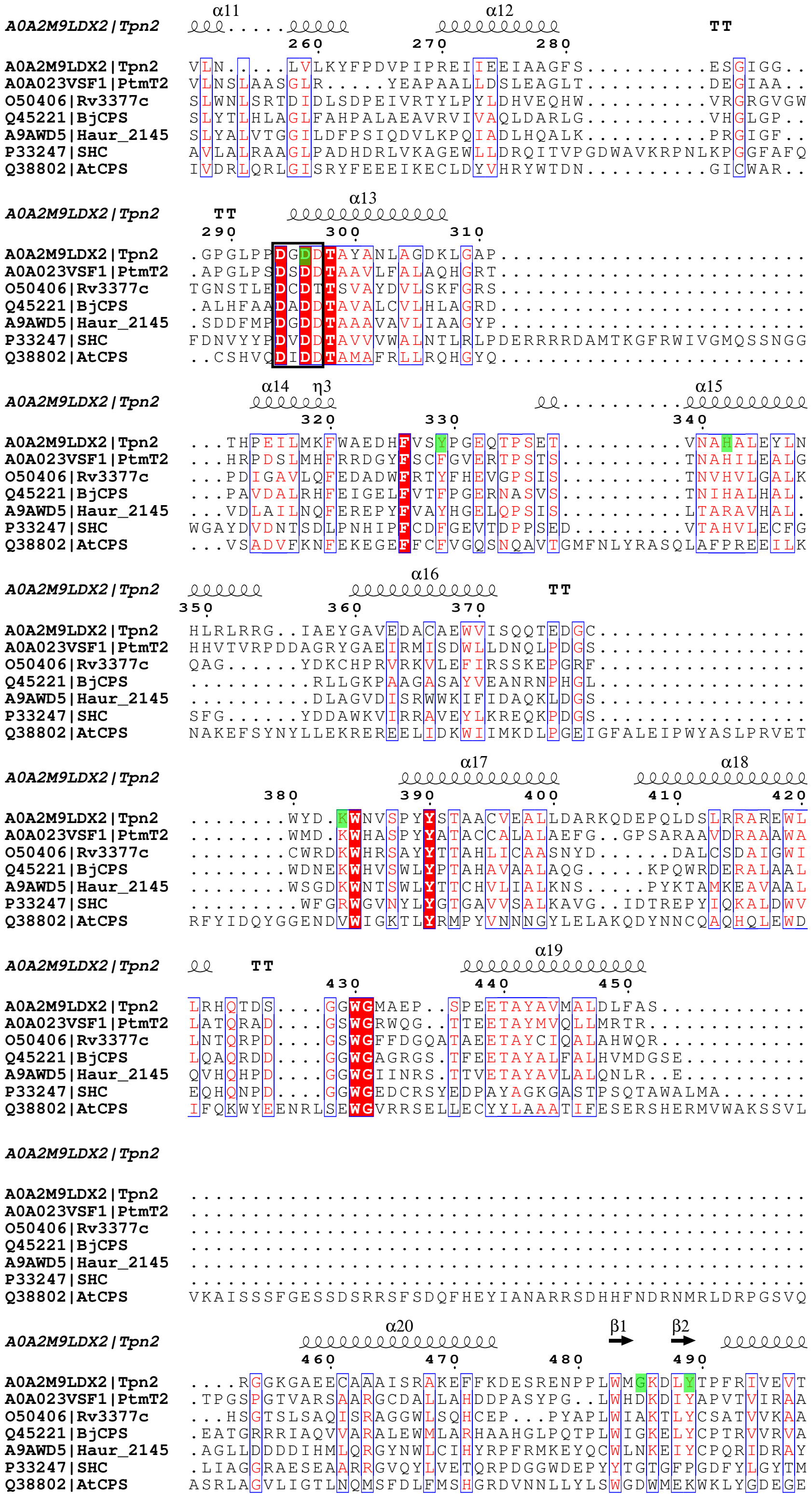

**Supplementary Fig. 6 cont.** Sequence alignment of selected bacterial and plant type II TSs.

```
                     α21
A0A2M9LDX2|Tpn2       ℓℓℓℓℓℓℓℓℓ
                     500
                        .
A0A2M9LDX2|Tpn2      VMCGRAVVSRY.......................................
A0A023VSF1|PtmT2     RLAALALGGAASAASGGA................................
O50406|Rv3377c       ILSALRLVDESNQ.....................................
Q45221|BjCPS         ELAGLWLALRWGRRVLAEGAGAAP..........................
A9AWD5|Haur_2145     ELS.AMLAVTLGELKL..................................
P33247|SHC           YRHVFPTLALGRYKQAIERR..............................
Q38802|AtCPS         GELMVKMIILMKNNDLTNFFTHTHFVRLAEIINRICLPRQYLKARRNDEK

A0A2M9LDX2|Tpn2

A0A2M9LDX2|Tpn2      ..................................................
A0A023VSF1|PtmT2     ..................................................
O50406|Rv3377c       ..................................................
Q45221|BjCPS         ..................................................
A9AWD5|Haur_2145     ..................................................
P33247|SHC           ..................................................
Q38802|AtCPS         EKTIKSMEKEMGKMVELALSESDTFRDVSITFLDVAKAFYYFALCGDHLQ

A0A2M9LDX2|Tpn2

A0A2M9LDX2|Tpn2      ...........
A0A023VSF1|PtmT2     ...........
O50406|Rv3377c       ...........
Q45221|BjCPS         ...........
A9AWD5|Haur_2145     ...........
P33247|SHC           ...........
Q38802|AtCPS         THISKVLFQKV
```

**Supplementary Fig. 7.** HPLC chromatograms showing in vitro enzyme reactions of Tpn2 and mutants after desphosphorylation.

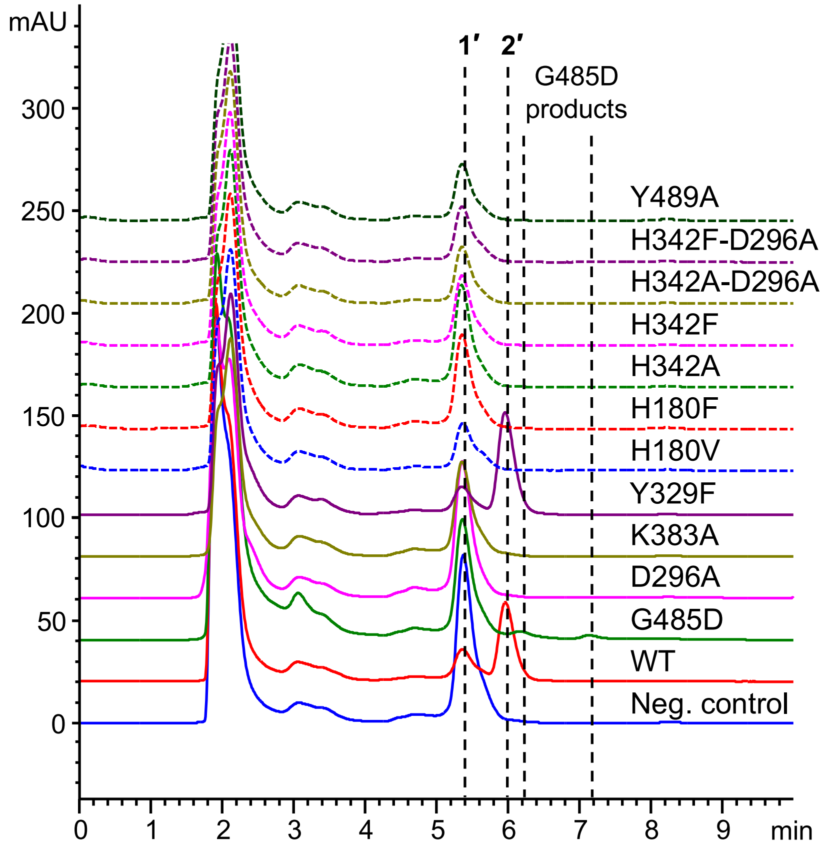

**Supplementary Fig. 8.** Scheme of the in vivo terpenoid upregulation system used in *E. coli* for heterologous production of Tpn2 products.[3]

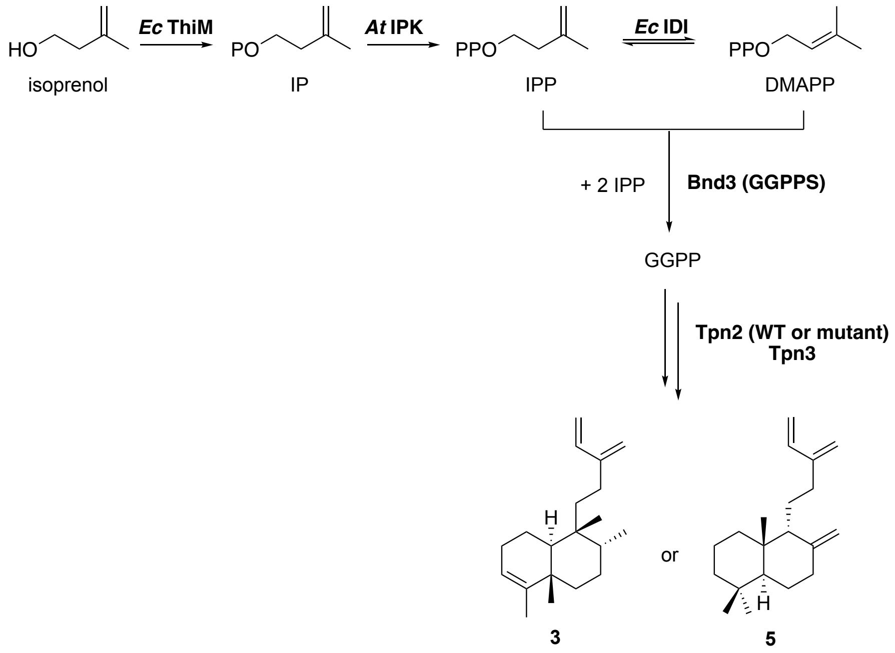

**Supplementary Fig. 9.** HPLC analysis showing production of terpentetriene (**3**) in vivo using the MKI4 system. The acyclic compounds **6** and **7** are produced via diphosphate elimination of GGPP by Tpn3.

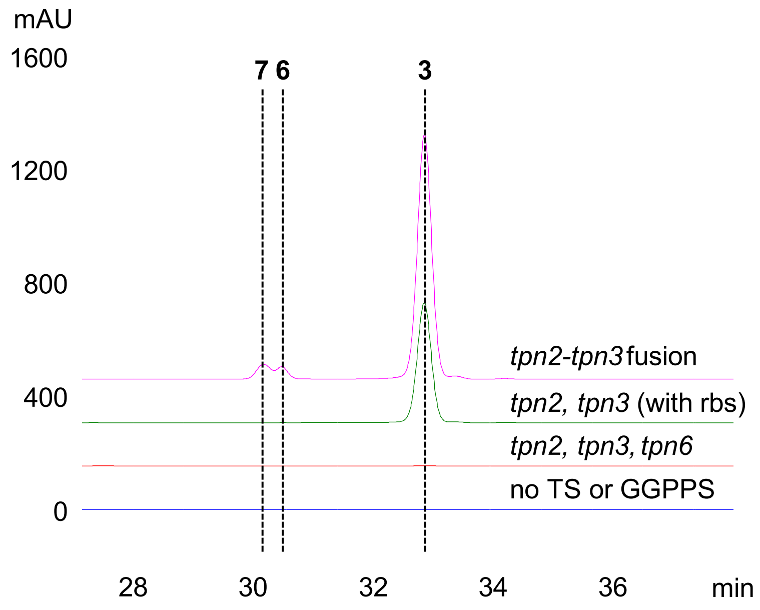

**Supplementary Fig. 10.** $^{1}$H NMR spectrum of terpentetriene (**3**) in $CDCl_3$ (600 MHz).

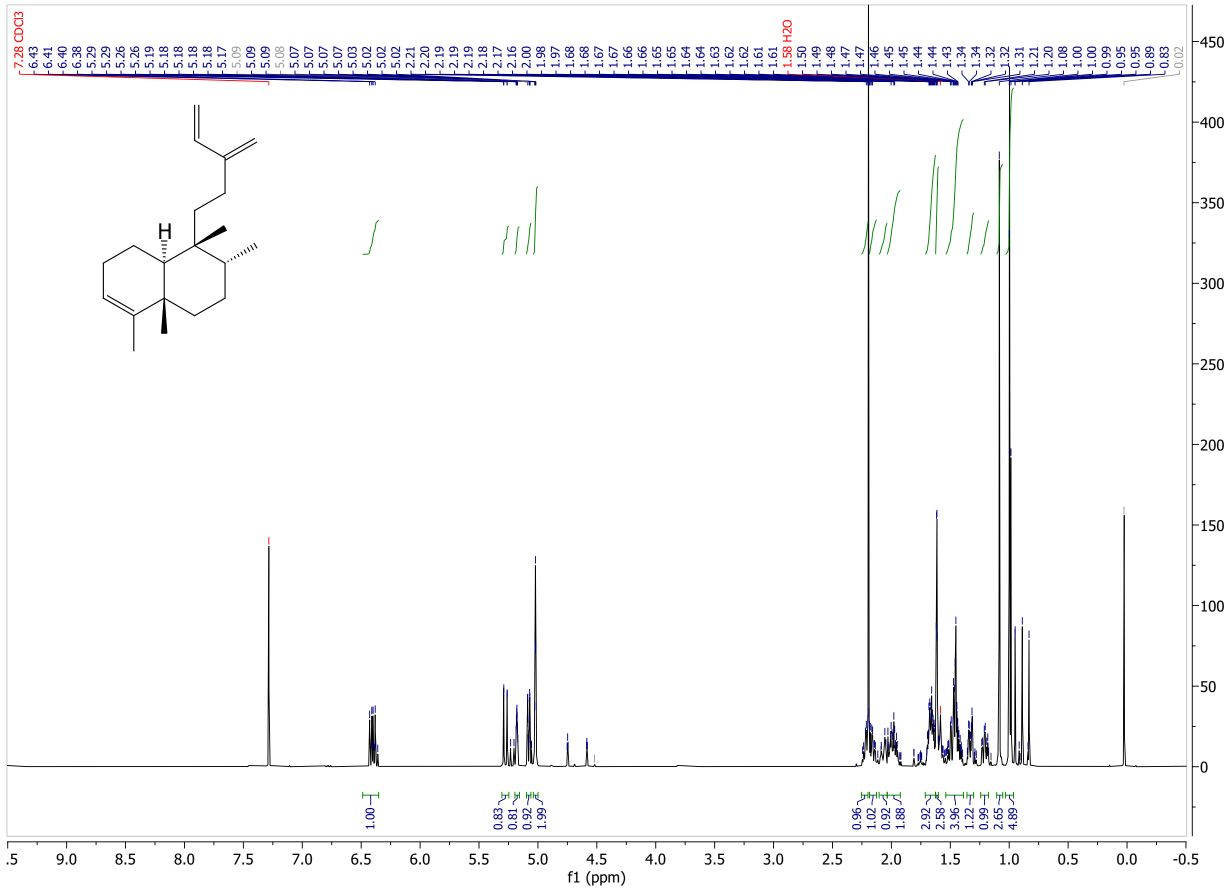

**Supplementary Fig. 11.** $^{13}C$ NMR spectrum of terpentetriene (**3**) in $CDCl_3$ (151 MHz).

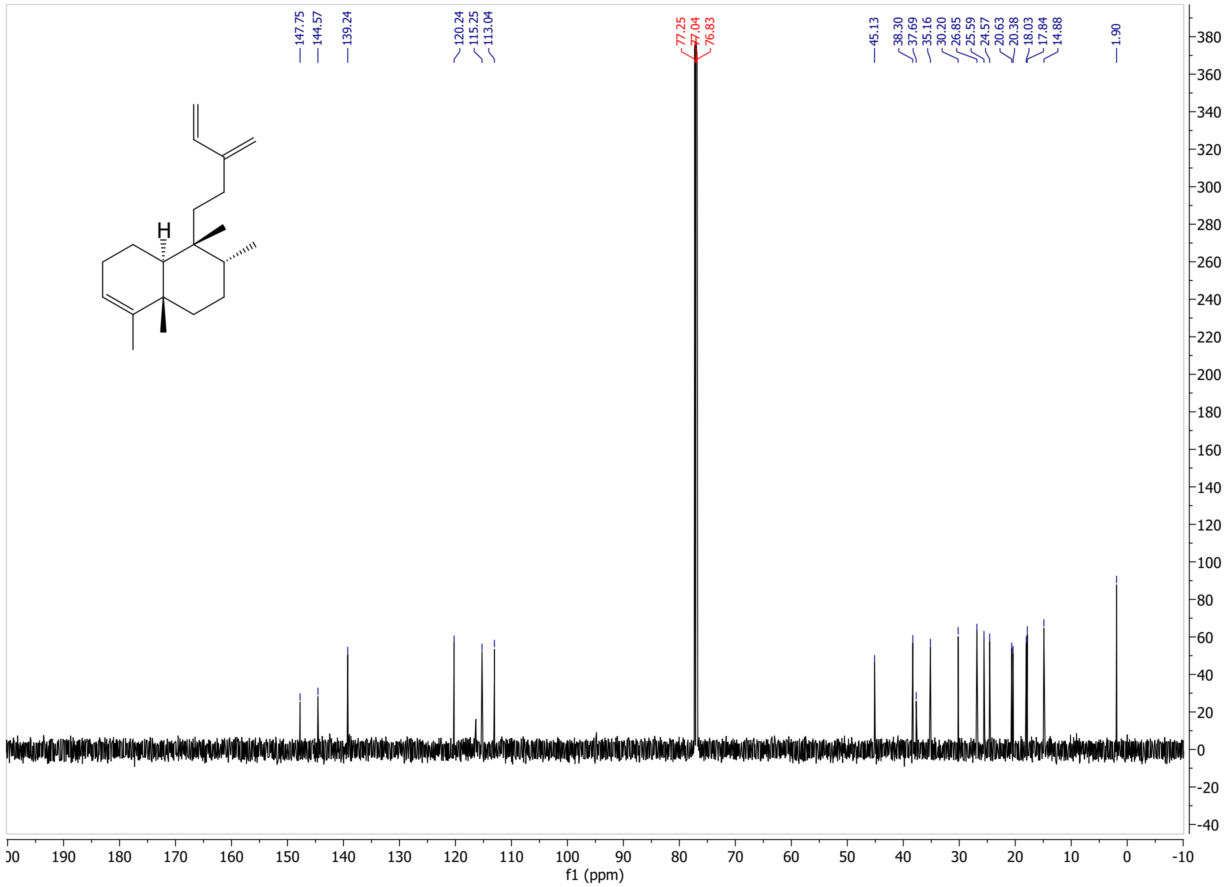

**Supplementary Fig. 12.** GC-MS analysis of in vivo products from Tpn2-Tpn3 and Tpn2$^{G485D}$-Tpn3 fusion proteins using the MKI4 system. Extracts of Tpn2-Tpn3 (a) and Tpn2$^{G485D}$-Tpn3 (b) revealed the production of major products **3** and **5**, respectively. The acyclic compounds **6** and **7** are produced via diphosphate elimination of GGPP by Tpn3. Two additional compounds produced by Tpn2$^{G485D}$-Tpn3 are proposed to be diterpene-related based on their *m/z* values of 272 and 290 and comparison to the NIST database (NIST Mass Spectral Search Program for the NIST/EPA/NIH Mass Spectral Library, Version 2.3, build May 4, 2017); the titers of these compounds were too low for isolation.

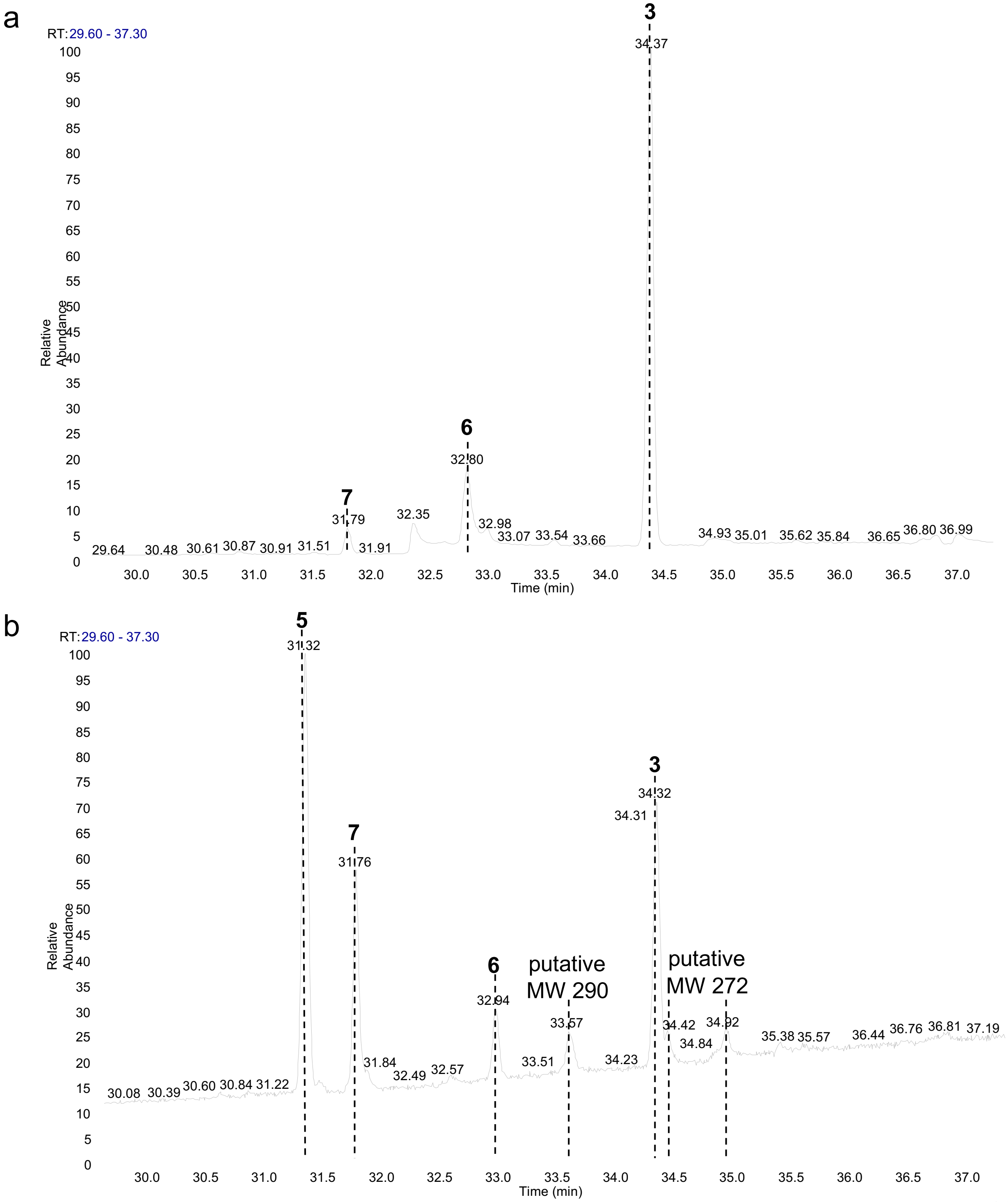

**Supplementary Fig. 13.** $^{1}$H NMR spectrum of α-springene (**6**) in $CDCl_3$ (600 MHz).

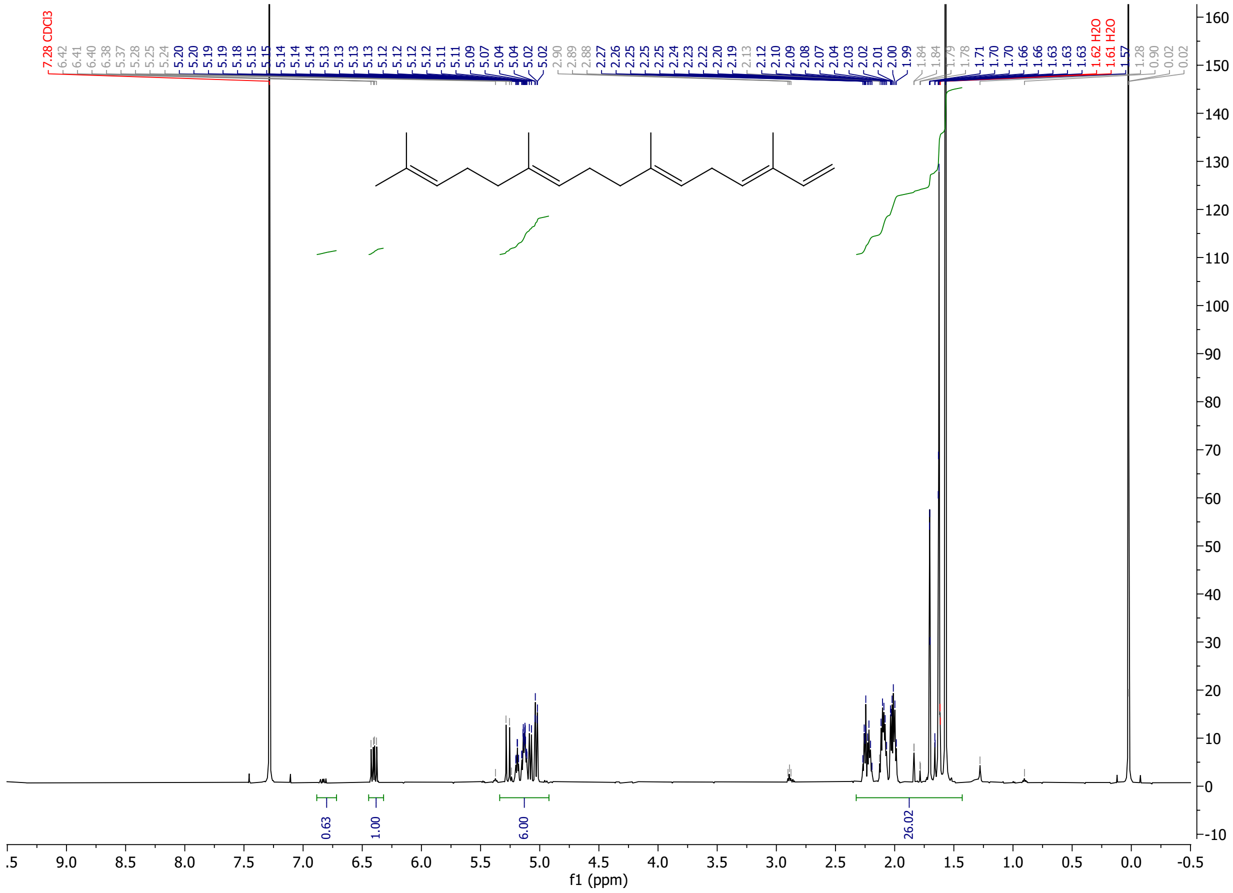

**Supplementary Fig. 14.** $^{1}$H NMR spectrum of β-springene (**7**) in $CDCl_3$ (600 MHz).

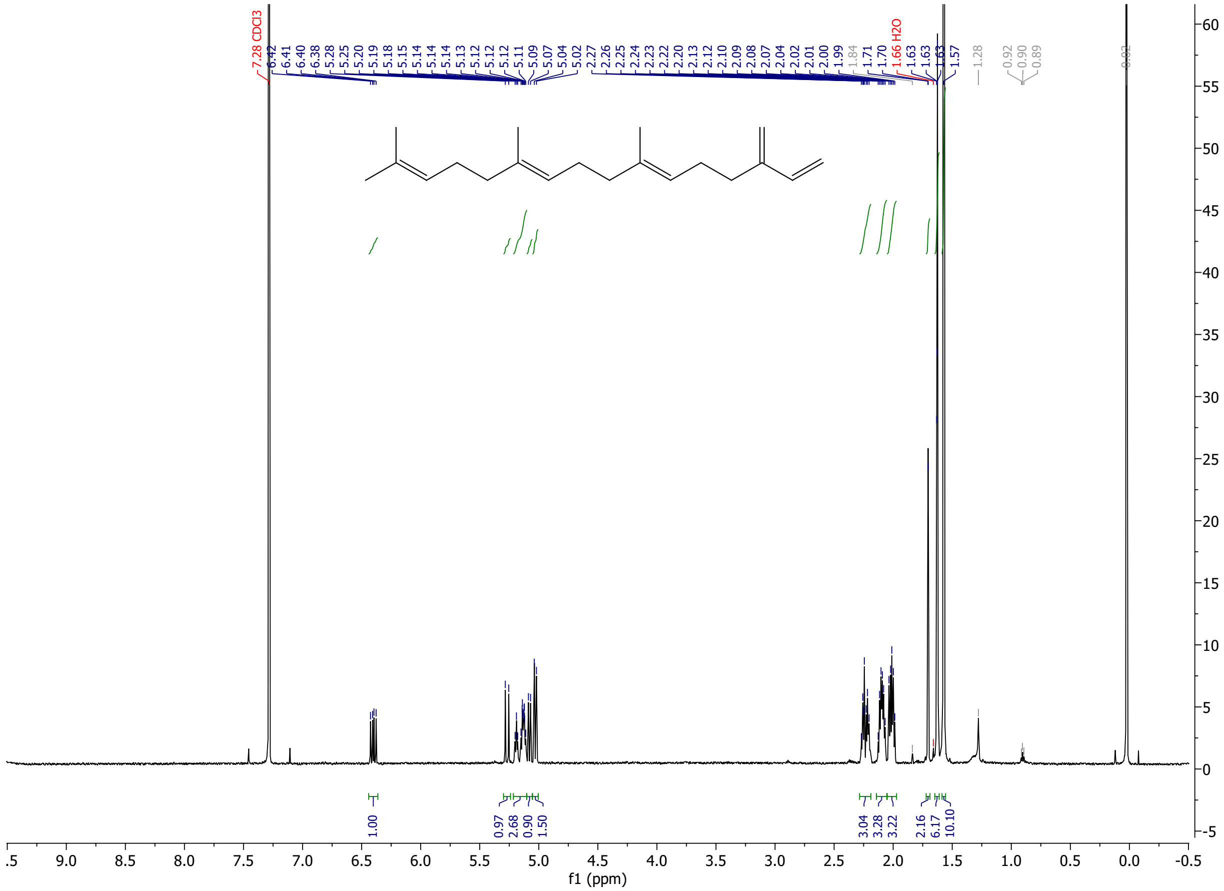

**Supplementary Fig. 15.** $^{1}H$ NMR spectrum of *syn*-sclarene (**5**) in $CDCl_3$ (600 MHz).

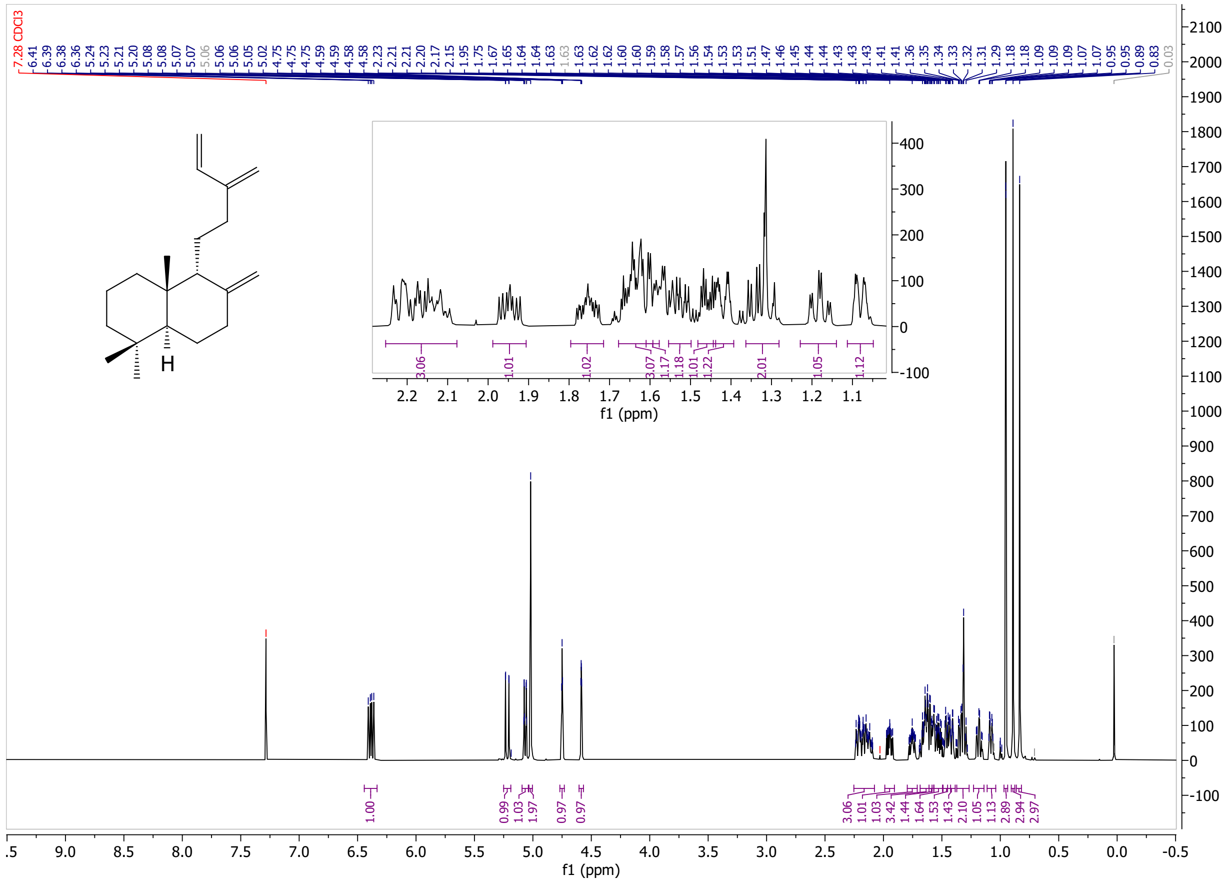

**Supplementary Fig. 16.** $^{13}C$ NMR spectrum of *syn*-sclarene (**5**) in $CDCl_3$ (151 MHz).

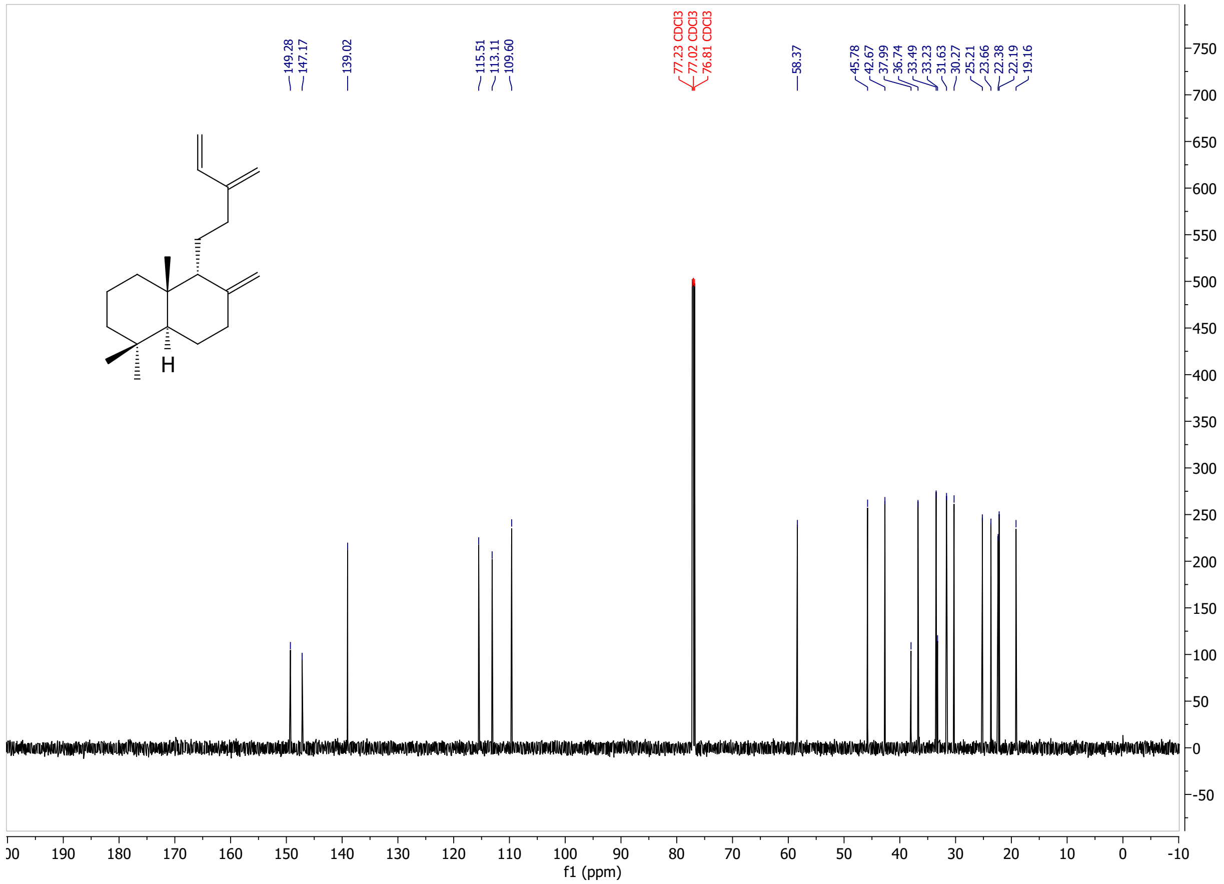

**Supplementary Fig. 17.** HSQC spectrum of *syn*-sclarene (**5**) in $CDCl_3$. Blue cross peaks represent –CH or –$CH_3$; orange represent –$CH_2$.

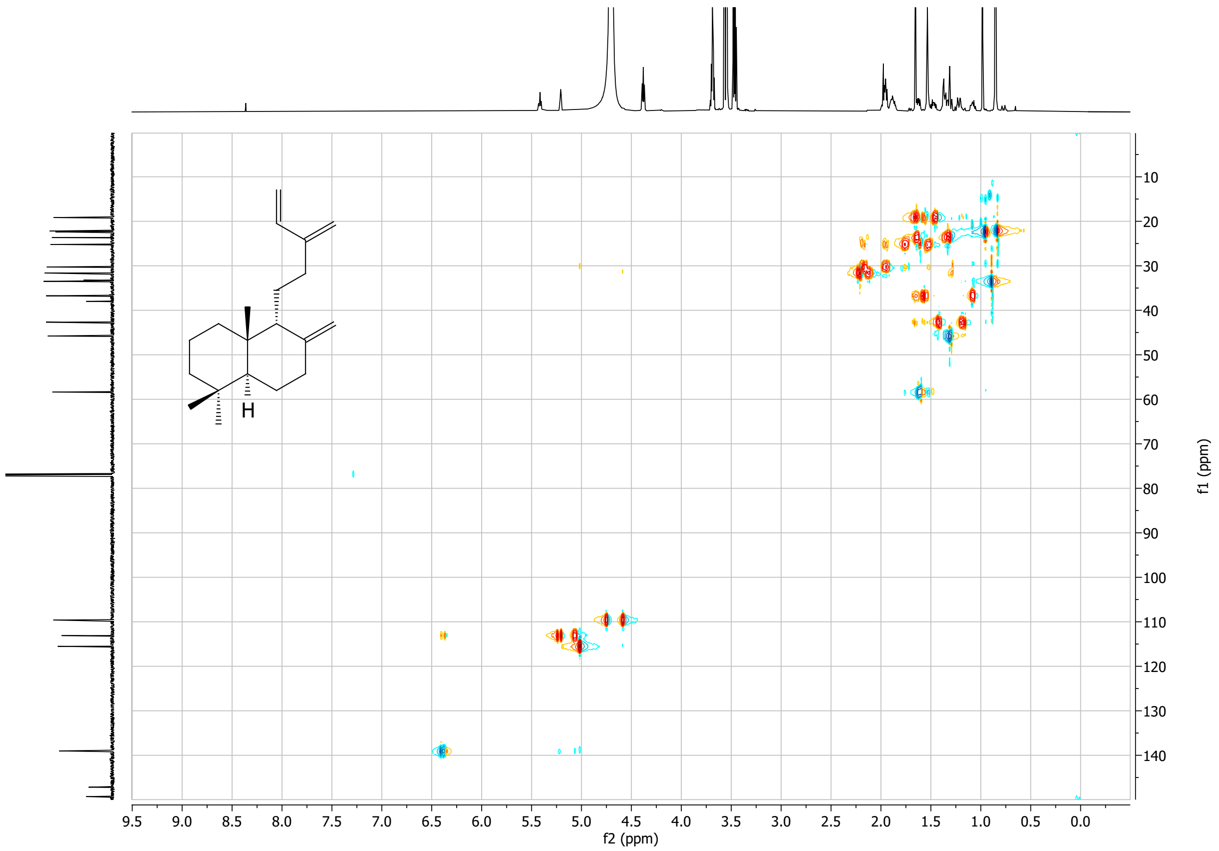

**Supplementary Fig. 18.** HMBC spectrum of *syn*-sclarene (**5**) in $CDCl_3$.

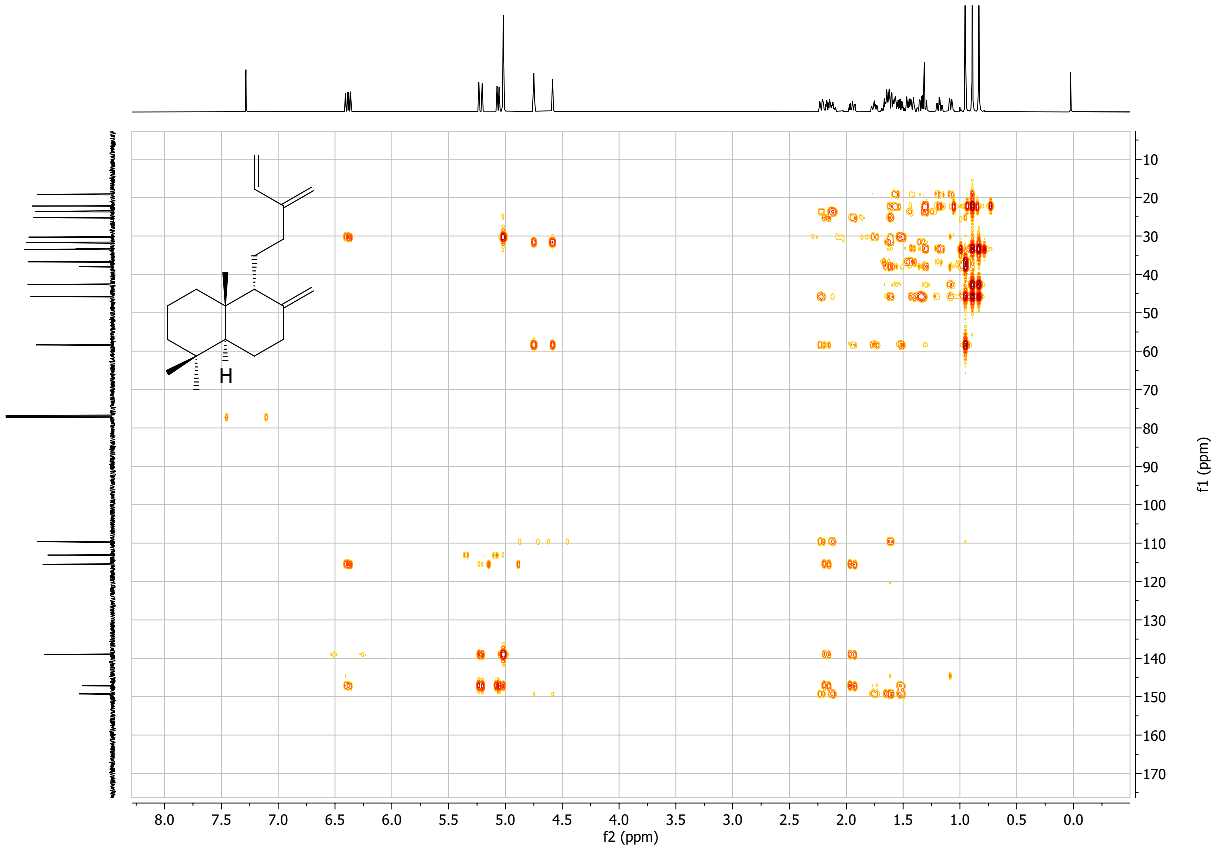

**Supplementary Fig. 19.** COSY spectrum of *syn*-sclarene (**5**) in $CDCl_3$.

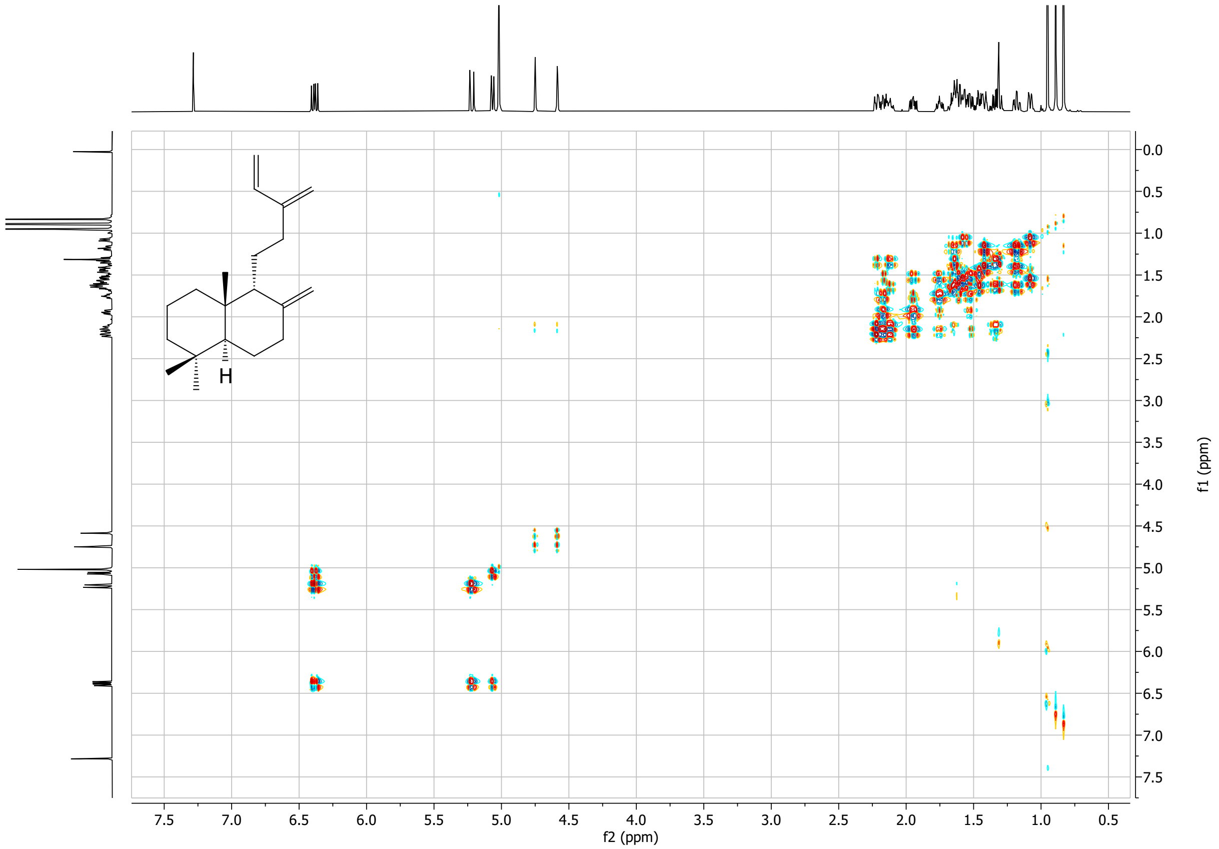

**Supplementary Fig. 20**. 2D NOESY spectrum of *syn*-sclarene (**5**) in $CDCl_3$ with key NOE correlations shown.

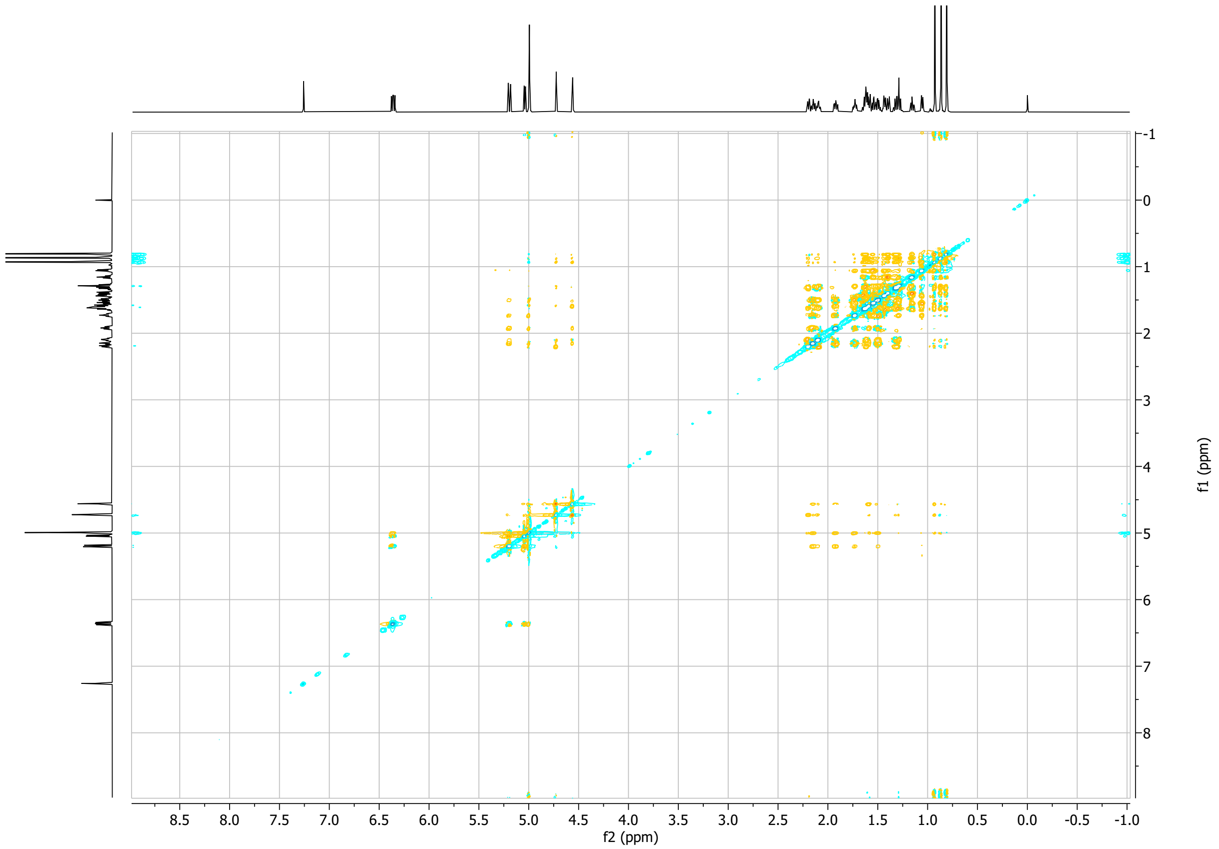

**Supplementary Fig. 21.** HPLC analysis of in vivo products from Tpn2$^{G485E}$-Tpn3 fusion protein using the MKI4 system.

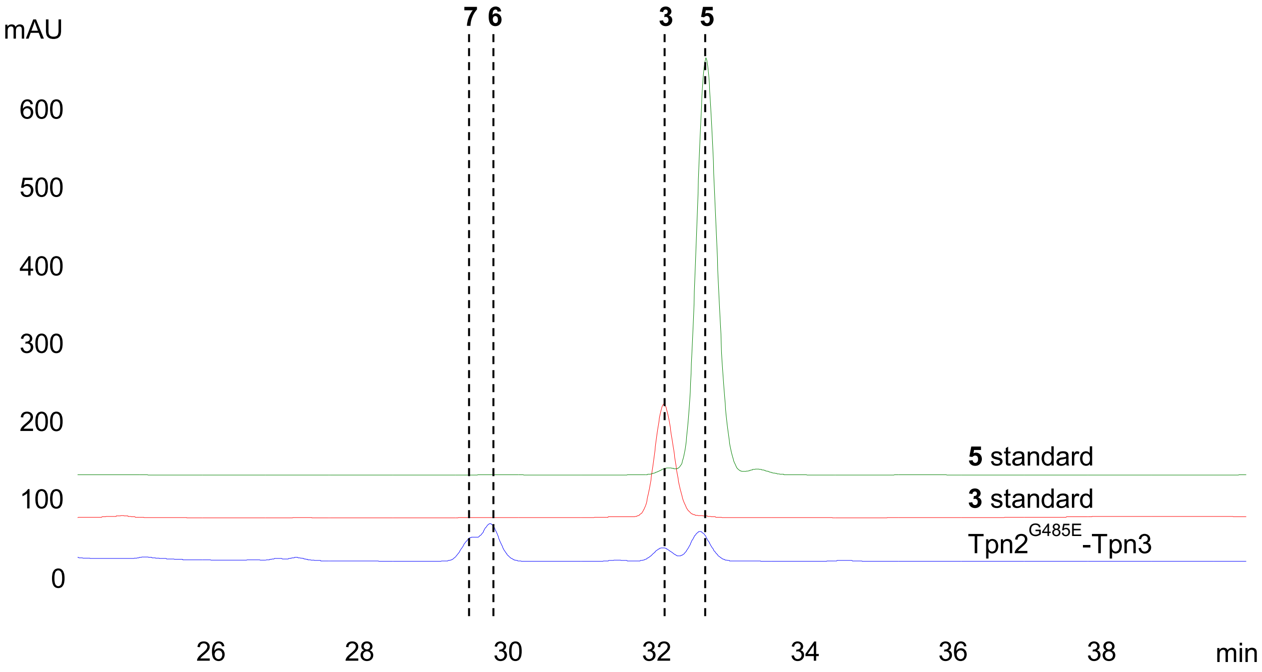

**Supplementary Fig. 22.** Genome neighborhood network of bacterial type II TSs.

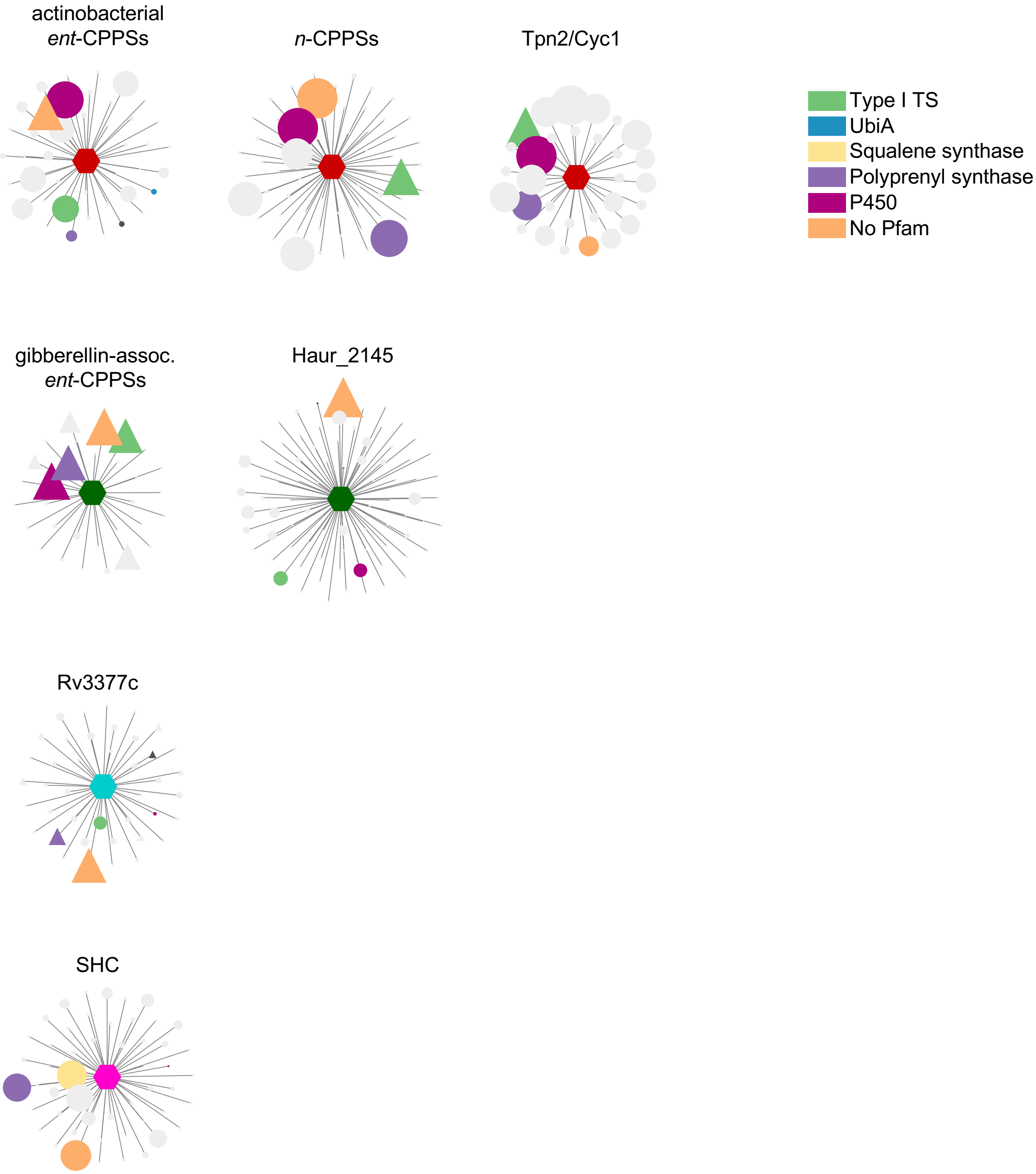

**Supplementary References**


1 Pan, G., et al., Discovery of the leinamycin family of natural products by mining actinobacterial genomes. *Proc. Natl. Acad. Sci. U. S. A*. **114**, E11131–E11140 (2017).

2 Lohman, J. R., Bingman, C. A., Phillips, G. N. & Shen, B., Structure of the bifunctional acyltransferase/decarboxylase LnmK from the leinamycin biosynthetic pathway revealing novel activity for a double-hot-dog fold. *Biochemistry* **52**, 902–911 (2013).

3 Xu, B., Ning, W., Wei, X. & Rudolf, J. D., Mutation of the eunicellane synthase Bnd4 alters its product profile and expands its prenylation ability. Preprint at 10.26434/chemrxiv-2022-3vsmz (2022).

4 Thompson, J. D., Higgins, D. G. & Gibosn, T. J., CLUSTAL W: improving the sensitivity of progressive multiple sequence alignment through sequence weighting, position-specific gap penalties and weight matrix choice. *Nucleic Acids Res.* **22**, 4673–4680 (1994).

5 Robert, X. & Gouet, P., Deciphering key features in protein structures with the new ENDscript server. *Nucleic Acids Res.* **42**, W320–W324 (2014).
