# Supplementary material for: Structure-guided product determination of the bacterial type II diterpene synthase Tpn2
Source: Commun Chem. 2022 Nov 8;5:146. doi: 10.1038/s42004-022-00765-6 (PMC9814783; doi:10.1038/s42004-022-00765-6)
Supplement: Supplementary file 3 — Description of Additional Supplementary Files [file 42004_2022_765_MOESM3_ESM.pdf]

Description of Additional Supplementary Files

File name: Supplementary Data 1

Description: PDB file

File name: Supplementary Data 2

Description: PDB Validation Report
