# Supplementary material for: Structure-guided product determination of the bacterial type II diterpene synthase Tpn2
Source: Commun Chem. 2022 Nov 8;5:146. doi: 10.1038/s42004-022-00765-6 (PMC9814783; doi:10.1038/s42004-022-00765-6)
Supplement: Supplementary file 5 — Supplementary Data 2 [file 42004_2022_765_MOESM5_ESM.pdf]

Full wwPDB X-ray Structure Validation Report (i)

Sep 20, 2022 – 09:12 AM JST

PDB ID	:	7XKX
Title	:	Crystal structure of Tpn2
Deposited on	:	2022-04-20
Resolution	:	2.57 Å(reported)

This wwPDB validation report is for manuscript review

For Manuscript Review

This is a Full wwPDB X-ray Structure Validation Report.

This report is produced by the wwPDB biocuration pipeline after annotation of the structure.

We welcome your comments at *validation@mail.wwpdb.org*
A user guide is available at
https://www.wwpdb.org/validation/2017/XrayValidationReportHelp
with specific help available everywhere you see the (i) symbol.

The types of validation reports are described at http://www.wwpdb.org/validation/2017/FAQs#types.

The following versions of software and data (see references (i)) were used in the production of this report:

MolProbity	:	4.02b-467
Xtriage (Phenix)	:	1.13
EDS	:	2.30
Percentile statistics	:	20191225.v01 (using entries in the PDB archive December 25th 2019)
Refmac	:	5.8.0158
CCP4	:	7.0.044 (Gargrove)
Ideal geometry (proteins)	:	Engh & Huber (2001)
Ideal geometry (DNA, RNA)	:	Parkinson et al. (1996)
Validation Pipeline (wwPDB-VP)	:	2.30

1 Overall quality at a glance (i)

The following experimental techniques were used to determine the structure:
X-RAY DIFFRACTION

The reported resolution of this entry is 2.57 Å.

Percentile scores (ranging between 0-100) for global validation metrics of the entry are shown in the following graphic. The table shows the number of entries on which the scores are based.

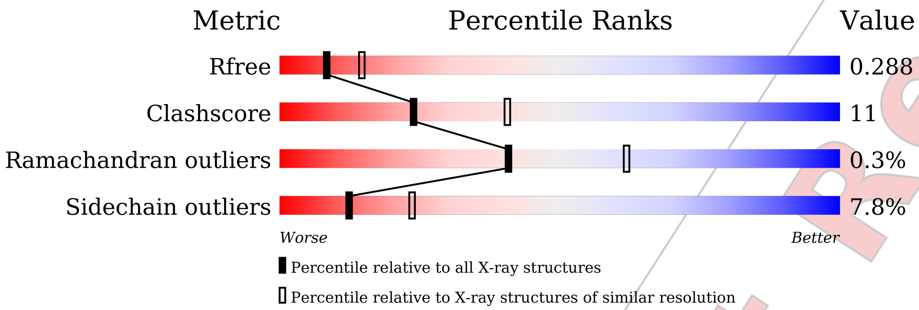

Metric	Whole archive (#Entries)	Similar resolution (#Entries, resolution range(Å))
R_{free}	130704	3676 (2.60-2.56)
Clashscore	141614	4049 (2.60-2.56)
Ramachandran outliers	138981	3979 (2.60-2.56)
Sidechain outliers	138945	3979 (2.60-2.56)

The table below summarises the geometric issues observed across the polymeric chains and their fit to the electron density. The red, orange, yellow and green segments of the lower bar indicate the fraction of residues that contain outliers for >=3, 2, 1 and 0 types of geometric quality criteria respectively. A grey segment represents the fraction of residues that are not modelled. The numeric value for each fraction is indicated below the corresponding segment, with a dot representing fractions <=5%

Mol	Chain	Length	Quality of chain
1	A	532	66% 24% • 8%
1	B	532	65% 22% • 10%

For Manuscript Review

2 Entry composition (i)

There are 2 unique types of molecules in this entry. The entry contains 7603 atoms, of which 0 are hydrogens and 0 are deuteriums.

In the tables below, the ZeroOcc column contains the number of atoms modelled with zero occupancy, the AltConf column contains the number of residues with at least one atom in alternate conformation and the Trace column contains the number of residues modelled with at most 2 atoms.

- Molecule 1 is a protein called SQHop_cyclase_C domain-containing protein.

Mol	Chain	Residues	Atoms					ZeroOcc	AltConf	Trace
1	A	489	Total 3762	C 2382	N 646	O 719	S 15	0	0	0
1	B	478	Total 3681	C 2332	N 628	O 707	S 14	0	0	0

There are 46 discrepancies between the modelled and reference sequences:

Chain	Residue	Modelled	Actual	Comment	Reference
A	-22	MET	-	initiating methionine	UNP A0A2M9LDX2
A	-21	GLY	-	expression tag	UNP A0A2M9LDX2
A	-20	SER	-	expression tag	UNP A0A2M9LDX2
A	-19	SER	-	expression tag	UNP A0A2M9LDX2
A	-18	HIS	-	expression tag	UNP A0A2M9LDX2
A	-17	HIS	-	expression tag	UNP A0A2M9LDX2
A	-16	HIS	-	expression tag	UNP A0A2M9LDX2
A	-15	HIS	-	expression tag	UNP A0A2M9LDX2
A	-14	HIS	-	expression tag	UNP A0A2M9LDX2
A	-13	HIS	-	expression tag	UNP A0A2M9LDX2
A	-12	SER	-	expression tag	UNP A0A2M9LDX2
A	-11	GLN	-	expression tag	UNP A0A2M9LDX2
A	-10	ASP	-	expression tag	UNP A0A2M9LDX2
A	-9	PRO	-	expression tag	UNP A0A2M9LDX2
A	-8	GLY	-	expression tag	UNP A0A2M9LDX2
A	-7	ASP	-	expression tag	UNP A0A2M9LDX2
A	-6	GLU	-	expression tag	UNP A0A2M9LDX2
A	-5	ASN	-	expression tag	UNP A0A2M9LDX2
A	-4	LEU	-	expression tag	UNP A0A2M9LDX2
A	-3	TYR	-	expression tag	UNP A0A2M9LDX2
A	-2	PHE	-	expression tag	UNP A0A2M9LDX2
A	-1	GLN	-	expression tag	UNP A0A2M9LDX2
A	0	SER	-	expression tag	UNP A0A2M9LDX2
B	-22	MET	-	initiating methionine	UNP A0A2M9LDX2
B	-21	GLY	-	expression tag	UNP A0A2M9LDX2

Continued on next page...

For Manuscript Review

Continued from previous page...

Chain	Residue	Modelled	Actual	Comment	Reference
B	-20	SER	-	expression tag	UNP A0A2M9LDX2
B	-19	SER	-	expression tag	UNP A0A2M9LDX2
B	-18	HIS	-	expression tag	UNP A0A2M9LDX2
B	-17	HIS	-	expression tag	UNP A0A2M9LDX2
B	-16	HIS	-	expression tag	UNP A0A2M9LDX2
B	-15	HIS	-	expression tag	UNP A0A2M9LDX2
B	-14	HIS	-	expression tag	UNP A0A2M9LDX2
B	-13	HIS	-	expression tag	UNP A0A2M9LDX2
B	-12	SER	-	expression tag	UNP A0A2M9LDX2
B	-11	GLN	-	expression tag	UNP A0A2M9LDX2
B	-10	ASP	-	expression tag	UNP A0A2M9LDX2
B	-9	PRO	-	expression tag	UNP A0A2M9LDX2
B	-8	GLY	-	expression tag	UNP A0A2M9LDX2
B	-7	ASP	-	expression tag	UNP A0A2M9LDX2
B	-6	GLU	-	expression tag	UNP A0A2M9LDX2
B	-5	ASN	-	expression tag	UNP A0A2M9LDX2
B	-4	LEU	-	expression tag	UNP A0A2M9LDX2
B	-3	TYR	-	expression tag	UNP A0A2M9LDX2
B	-2	PHE	-	expression tag	UNP A0A2M9LDX2
B	-1	GLN	-	expression tag	UNP A0A2M9LDX2
B	0	SER	-	expression tag	UNP A0A2M9LDX2

- Molecule 2 is water.

Mol	Chain	Residues	Atoms	ZeroOcc	AltConf
2	A	94	Total O<br>94 94	0	0
2	B	66	Total O<br>66 66	0	0

For Manuscript Review

3 Residue-property plots (i)

These plots are drawn for all protein, RNA, DNA and oligosaccharide chains in the entry. The first graphic for a chain summarises the proportions of the various outlier classes displayed in the second graphic. The second graphic shows the sequence view annotated by issues in geometry. Residues are color-coded according to the number of geometric quality criteria for which they contain at least one outlier: green = 0, yellow = 1, orange = 2 and red = 3 or more. Stretches of 2 or more consecutive residues without any outlier are shown as a green connector. Residues present in the sample, but not in the model, are shown in grey.

- Molecule 1: SQHop_cyclase_C domain-containing protein

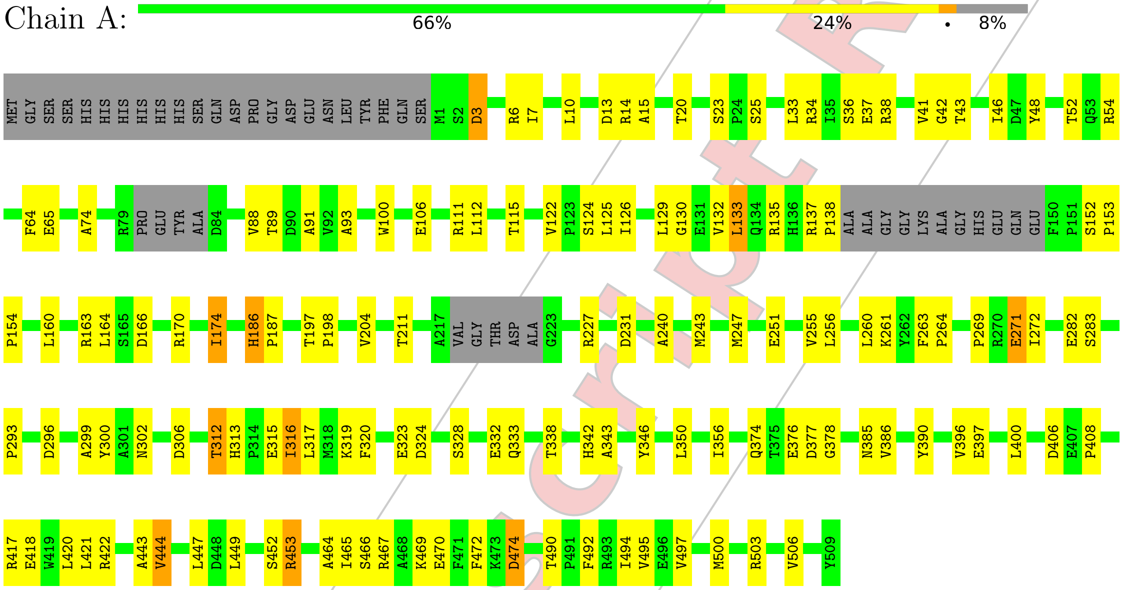

- Molecule 1: SQHop_cyclase_C domain-containing protein

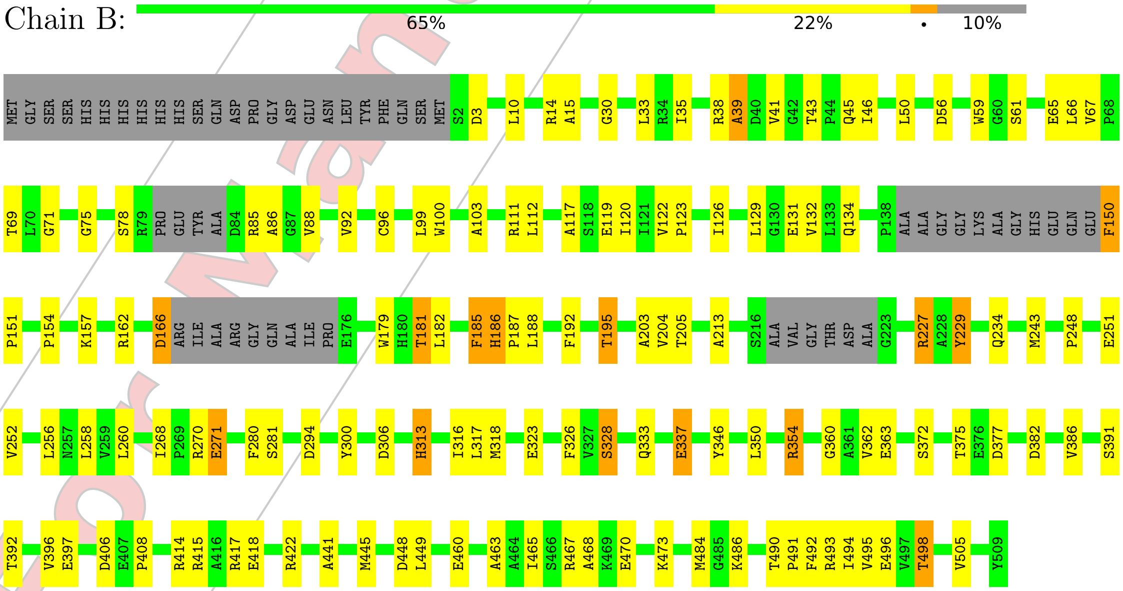

For Manuscript Review

4 Data and refinement statistics (i)

Property	Value	Source
Space group	P 31	Depositor
Cell constants<br>a, b, c, α, β, γ	92.54Å 92.54Å 127.91Å<br>90.00° 90.00° 120.00°	Depositor
Resolution (Å)	27.39 – 2.57<br>27.38 – 2.57	Depositor<br>EDS
% Data completeness<br>(in resolution range)	99.2 (27.39-2.57)<br>99.3 (27.38-2.57)	Depositor<br>EDS
R_{merge}	0.12	Depositor
R_{sym}	(Not available)	Depositor
$< I/\sigma(I) >$ [1]	3.41 (at 2.57Å)	Xtriage
Refinement program	REFMAC 5.8.0267	Depositor
R, R_{free}	0.253 , 0.288<br>0.256 , 0.288	Depositor<br>DCC
R_{free} test set	2048 reflections (5.29%)	wwPDB-VP
Wilson B-factor (Å^2)	52.6	Xtriage
Anisotropy	0.013	Xtriage
Bulk solvent k_{sol}(e/Å^3), B_{sol}(Å^2)	0.28 , 24.7	EDS
L-test for twinning[2]	$< \|L\| >$ = 0.40, $< L^2 >$ = 0.22	Xtriage
Estimated twinning fraction	0.320 for -h,-k,l<br>0.104 for h,-h-k,-l<br>0.098 for -k,-h,-l	Xtriage
F_o,F_c correlation	0.94	EDS
Total number of atoms	7603	wwPDB-VP
Average B, all atoms (Å^2)	57.0	wwPDB-VP

Xtriage's analysis on translational NCS is as follows: *The largest off-origin peak in the Patterson function is 2.46% of the height of the origin peak. No significant pseudotranslation is detected.*

[1] Intensities estimated from amplitudes.
[2] Theoretical values of $< |L| >$, $< L^2 >$ for acentric reflections are 0.5, 0.333 respectively for untwinned datasets, and 0.375, 0.2 for perfectly twinned datasets.

For Manuscript Review

5 Model quality (i)

5.1 Standard geometry (i)

The Z score for a bond length (or angle) is the number of standard deviations the observed value is removed from the expected value. A bond length (or angle) with $|Z| > 5$ is considered an outlier worth inspection. RMSZ is the root-mean-square of all Z scores of the bond lengths (or angles).

Mol	Chain	Bond lengths		Bond angles	
		RMSZ	#|Z| >5	RMSZ	#|Z| >5
1	A	0.64	0/3861	0.78	0/5263
1	B	0.65	0/3778	0.75	0/5150
All	All	0.65	0/7639	0.77	0/10413

There are no bond length outliers.

There are no bond angle outliers.

There are no chirality outliers.

There are no planarity outliers.

5.2 Too-close contacts (i)

In the following table, the Non-H and H(model) columns list the number of non-hydrogen atoms and hydrogen atoms in the chain respectively. The H(added) column lists the number of hydrogen atoms added and optimized by MolProbity. The Clashes column lists the number of clashes within the asymmetric unit, whereas Symm-Clashes lists symmetry-related clashes.

Mol	Chain	Non-H	H(model)	H(added)	Clashes	Symm-Clashes
1	A	3762	0	3639	83	0
1	B	3681	0	3545	82	0
2	A	94	0	0	0	0
2	B	66	0	0	1	0
All	All	7603	0	7184	163	0

The all-atom clashscore is defined as the number of clashes found per 1000 atoms (including hydrogen atoms). The all-atom clashscore for this structure is 11.

All (163) close contacts within the same asymmetric unit are listed below, sorted by their clash magnitude.

Atom-1	Atom-2	Interatomic distance (Å)	Clash overlap (Å)
1:B:119:GLU:HB2	1:B:157:LYS:HB2	1.48	0.93

Continued on next page...

For Manuscript Review

Continued from previous page...

Atom-1	Atom-2	Interatomic distance (Å)	Clash overlap (Å)
1:A:20:THR:HG21	1:A:247:MET:H	1.43	0.82
1:B:494:ILE:O	1:B:498:THR:HG23	1.78	0.82
1:B:112:LEU:HD21	1:B:122:VAL:HG21	1.64	0.78
1:A:377:ASP:HB2	1:B:38:ARG:HD2	1.67	0.76
1:A:137:ARG:HG2	1:A:138:PRO:HD2	1.69	0.74
1:A:137:ARG:CG	1:A:138:PRO:HD2	2.19	0.72
1:B:243:MET:O	1:B:243:MET:HE3	1.87	0.72
1:A:472:PHE:HB3	1:A:503:ARG:HD3	1.72	0.71
1:A:34:ARG:HH12	1:A:186:HIS:HB3	1.55	0.70
1:A:251:GLU:HA	1:A:494:ILE:HD11	1.72	0.70
1:A:418:GLU:HB3	1:A:422:ARG:HH21	1.55	0.69
1:B:418:GLU:OE2	1:B:422:ARG:NH2	2.25	0.68
1:A:374:GLN:NE2	1:A:378:GLY:O	2.28	0.67
1:B:46:ILE:O	1:B:50:LEU:HG	1.96	0.64
1:A:261:LYS:NZ	1:A:397:GLU:OE1	2.32	0.63
1:A:299:ALA:HB2	1:A:317:LEU:HD11	1.81	0.63
1:B:406:ASP:HB3	1:B:408:PRO:HD2	1.81	0.63
1:A:269:PRO:HG2	1:A:272:ILE:HD12	1.81	0.61
1:B:88:VAL:O	1:B:92:VAL:HG23	1.99	0.61
1:B:33:LEU:HD12	1:B:45:GLN:HB2	1.82	0.61
1:B:192:PHE:O	1:B:195:THR:HG22	2.03	0.59
1:B:134:GLN:HA	1:B:134:GLN:OE1	2.03	0.59
1:B:306:ASP:OD1	1:B:354:ARG:HD3	2.03	0.59
1:B:39:ALA:HB3	1:B:41:VAL:HG23	1.84	0.59
1:B:392:THR:O	1:B:396:VAL:HG23	2.02	0.59
1:A:490:THR:HG23	1:A:495:VAL:HG21	1.84	0.58
1:B:134:GLN:NE2	2:B:603:HOH:O	2.29	0.58
1:B:491:PRO:HB2	1:B:494:ILE:HD12	1.84	0.58
1:A:3:ASP:O	1:A:7:ILE:HD12	2.04	0.58
1:B:117:ALA:HB2	1:B:181:THR:HG21	1.84	0.58
1:B:490:THR:HG23	1:B:495:VAL:HG21	1.85	0.57
1:A:417:ARG:HG2	1:A:421:LEU:CD2	2.35	0.57
1:B:117:ALA:CB	1:B:181:THR:HG21	2.35	0.57
1:B:258:LEU:CD1	1:B:498:THR:HG22	2.35	0.57
1:B:463:ALA:O	1:B:467:ARG:HG3	2.04	0.57
1:B:181:THR:O	1:B:181:THR:OG1	2.22	0.56
1:A:6:ARG:O	1:A:10:LEU:HG	2.05	0.56
1:B:30:GLY:HA3	1:B:71:GLY:O	2.06	0.56
1:B:258:LEU:HD13	1:B:498:THR:HG22	1.88	0.56
1:B:441:ALA:O	1:B:445:MET:HG3	2.06	0.55
1:B:103:ALA:HB1	1:B:151:PRO:HG3	1.89	0.55

Continued on next page...

For Manuscript Review

Continued from previous page...

Atom-1	Atom-2	Interatomic distance (Å)	Clash overlap (Å)
1:A:112:LEU:CD2	1:A:122:VAL:HG21	2.37	0.55
1:B:179:TRP:HB3	1:B:205:THR:HG21	1.89	0.54
1:A:470:GLU:O	1:A:474:ASP:HB2	2.07	0.54
1:B:185:PHE:O	1:B:188:LEU:HD12	2.08	0.54
1:A:316:ILE:O	1:A:319:LYS:HB2	2.09	0.53
1:B:227:ARG:NH2	1:B:234:GLN:OE1	2.42	0.53
1:A:64:PHE:CE1	1:A:386:VAL:HG21	2.44	0.53
1:A:122:VAL:HG11	1:A:153:PRO:HG2	1.90	0.53
1:A:464:ALA:HA	1:A:467:ARG:HD2	1.90	0.53
1:A:125:LEU:O	1:A:129:LEU:HB2	2.09	0.53
1:A:112:LEU:HD21	1:A:122:VAL:HG21	1.90	0.53
1:A:74:ALA:HB2	1:A:129:LEU:HD11	1.91	0.53
1:A:20:THR:HG21	1:A:247:MET:N	2.20	0.52
1:B:111:ARG:HG3	1:B:154:PRO:HB2	1.90	0.52
1:B:360:GLY:O	1:B:363:GLU:HB2	2.09	0.52
1:A:198:PRO:HB3	1:A:204:VAL:HG12	1.91	0.52
1:A:15:ALA:HB1	1:A:271:GLU:HG3	1.92	0.52
1:B:397:GLU:HG2	1:B:449:LEU:CD1	2.40	0.52
1:B:166:ASP:N	1:B:166:ASP:OD1	2.42	0.51
1:B:375:THR:O	1:B:415:ARG:NH2	2.43	0.51
1:A:376:GLU:HB2	1:B:38:ARG:HH12	1.74	0.51
1:B:10:LEU:HD21	1:B:493:ARG:HG2	1.93	0.50
1:A:89:THR:O	1:A:93:ALA:N	2.38	0.50
1:B:85:ARG:HG3	1:B:86:ALA:H	1.77	0.50
1:A:313:HIS:O	1:A:316:ILE:HG22	2.11	0.50
1:B:33:LEU:HD22	1:B:75:GLY:O	2.11	0.50
1:B:179:TRP:O	1:B:182:LEU:HB2	2.12	0.50
1:A:256:LEU:O	1:A:260:LEU:HG	2.11	0.49
1:A:338:THR:HG21	1:A:390:TYR:CE2	2.47	0.49
1:B:35:ILE:HG21	1:B:213:ALA:HA	1.94	0.49
1:B:150:PHE:N	1:B:151:PRO:CD	2.75	0.49
1:A:204:VAL:HG13	1:A:211:THR:HG23	1.93	0.49
1:B:256:LEU:HD11	1:B:268:ILE:HG12	1.94	0.49
1:B:317:LEU:HG	1:B:326:PHE:CE1	2.47	0.49
1:B:112:LEU:CD2	1:B:122:VAL:HG21	2.39	0.49
1:B:15:ALA:HB1	1:B:271:GLU:HG3	1.94	0.49
1:B:67:VAL:HG11	1:B:486:LYS:O	2.12	0.49
1:A:7:ILE:HG13	1:A:500:MET:HE1	1.95	0.49
1:A:283:SER:HA	1:A:316:ILE:HD11	1.95	0.49
1:A:302:ASN:HB3	1:A:346:TYR:CE1	2.48	0.48
1:A:296:ASP:OD1	1:A:342:HIS:HD2	1.97	0.48

Continued on next page...

For Manuscript Review

Continued from previous page...

Atom-1	Atom-2	Interatomic distance (Å)	Clash overlap (Å)
1:B:123:PRO:HA	1:B:126:ILE:HG12	1.96	0.48
1:A:111:ARG:HA	1:A:154:PRO:HD2	1.96	0.48
1:A:186:HIS:HB2	1:A:187:PRO:HD3	1.96	0.48
1:A:37:GLU:HG2	1:A:38:ARG:H	1.78	0.48
1:A:25:SER:HB2	1:A:243:MET:HB2	1.96	0.47
1:A:306:ASP:OD2	1:A:312:THR:HG21	2.15	0.47
1:B:96:CYS:HA	1:B:99:LEU:HB2	1.97	0.47
1:A:133:LEU:HD13	1:A:133:LEU:HA	1.69	0.47
1:A:255:VAL:HG13	1:A:497:VAL:HG21	1.97	0.47
1:A:299:ALA:HB1	1:A:343:ALA:HA	1.97	0.47
1:B:186:HIS:HB3	1:B:187:PRO:HD3	1.97	0.46
1:B:162:ARG:HA	1:B:162:ARG:HD2	1.72	0.46
1:A:160:LEU:HD23	1:A:163:ARG:HH22	1.81	0.46
1:B:386:VAL:HG13	1:B:484:MET:HG2	1.97	0.46
1:A:306:ASP:CG	1:A:312:THR:HG21	2.36	0.46
1:A:385:ASN:HB3	1:A:390:TYR:CD2	2.51	0.46
1:A:444:VAL:HG22	1:A:465:ILE:HG23	1.98	0.46
1:A:449:LEU:O	1:A:453:ARG:HG3	2.16	0.46
1:A:7:ILE:HG13	1:A:500:MET:CE	2.46	0.45
1:B:418:GLU:HG2	1:B:422:ARG:NH1	2.31	0.45
1:B:100:TRP:HA	1:B:103:ALA:HB3	1.97	0.45
1:B:248:PRO:O	1:B:252:VAL:HG23	2.17	0.45
1:A:166:ASP:O	1:A:170:ARG:HG3	2.17	0.45
1:B:280:PHE:HZ	1:B:316:ILE:HG21	1.80	0.45
1:A:23:SER:HB3	1:A:492:PHE:CZ	2.52	0.45
1:A:126:ILE:O	1:A:130:GLY:N	2.45	0.45
1:B:337:GLU:HG3	1:B:382:ASP:HA	1.98	0.45
1:B:203:ALA:HA	1:B:229:TYR:CE2	2.52	0.45
1:B:382:ASP:HB2	1:B:391:SER:HB3	1.97	0.45
1:A:14:ARG:HA	1:A:14:ARG:HD2	1.84	0.45
1:B:256:LEU:O	1:B:260:LEU:HG	2.17	0.45
1:B:414:ARG:HA	1:B:417:ARG:NH1	2.32	0.45
1:B:14:ARG:HH22	1:B:248:PRO:HB3	1.82	0.45
1:B:14:ARG:NH1	1:B:251:GLU:OE1	2.50	0.45
1:B:256:LEU:CD1	1:B:268:ILE:HG12	2.47	0.45
1:A:33:LEU:HD21	1:A:46:ILE:HG13	1.98	0.44
1:A:418:GLU:HB3	1:A:422:ARG:NH2	2.28	0.44
1:B:204:VAL:HG12	1:B:205:THR:HG22	1.99	0.44
1:A:54:ARG:NH2	1:A:65:GLU:HG2	2.32	0.44
1:A:444:VAL:HG22	1:A:465:ILE:HG12	2.00	0.44
1:B:346:TYR:OH	1:B:350:LEU:HD11	2.18	0.44

Continued on next page...

For Manuscript Review

Continued from previous page...

Atom-1	Atom-2	Interatomic distance (Å)	Clash overlap (Å)
1:B:294:ASP:HA	1:B:328:SER:O	2.18	0.44
1:A:306:ASP:OD2	1:A:346:TYR:HE1	2.01	0.43
1:B:66:LEU:HD12	1:B:66:LEU:HA	1.85	0.43
1:B:318:MET:HE2	1:B:362:VAL:HG21	1.99	0.43
1:A:323:GLU:HG3	1:A:324:ASP:N	2.33	0.43
1:A:153:PRO:HA	1:A:154:PRO:HD3	1.92	0.43
1:A:129:LEU:HA	1:A:132:VAL:HB	2.00	0.43
1:A:396:VAL:O	1:A:400:LEU:HG	2.18	0.43
1:B:35:ILE:HG23	1:B:186:HIS:CE1	2.54	0.43
1:A:397:GLU:HG2	1:A:449:LEU:CD1	2.49	0.43
1:A:263:PHE:N	1:A:264:PRO:HD3	2.34	0.42
1:A:88:VAL:O	1:A:91:ALA:HB3	2.20	0.42
1:A:164:LEU:HB3	1:A:174:ILE:HD11	2.00	0.42
1:A:135:ARG:HD3	1:A:135:ARG:HA	1.85	0.42
1:A:293:PRO:HB2	1:A:320:PHE:HZ	1.85	0.42
1:B:448:ASP:HB2	1:B:505:VAL:HG11	2.02	0.42
1:B:280:PHE:CZ	1:B:316:ILE:HD13	2.55	0.41
1:A:227:ARG:NH1	1:A:231:ASP:OD1	2.52	0.41
1:A:443:ALA:O	1:A:447:LEU:HG	2.20	0.41
1:B:313:HIS:O	1:B:316:ILE:HG22	2.19	0.41
1:B:65:GLU:O	1:B:69:THR:HB	2.20	0.41
1:A:48:TYR:CE1	1:A:52:THR:HG21	2.55	0.41
1:B:182:LEU:HD12	1:B:182:LEU:HA	1.92	0.41
1:A:48:TYR:CG	1:A:240:ALA:HB2	2.56	0.41
1:A:299:ALA:CB	1:A:317:LEU:HD11	2.50	0.41
1:B:119:GLU:HG2	1:B:120:ILE:HD12	2.03	0.41
1:A:282:GLU:HG2	1:A:283:SER:N	2.35	0.40
1:A:315:GLU:HG3	1:A:316:ILE:N	2.37	0.40
1:A:417:ARG:O	1:A:420:LEU:HB2	2.22	0.40
1:A:466:SER:O	1:A:469:LYS:HB2	2.22	0.40
1:A:472:PHE:HD2	1:A:506:VAL:HG21	1.86	0.40
1:B:59:TRP:O	1:B:65:GLU:HA	2.21	0.40
1:B:99:LEU:HD23	1:B:99:LEU:HA	1.87	0.40
1:B:179:TRP:CB	1:B:205:THR:HG21	2.51	0.40
1:A:122:VAL:CG1	1:A:153:PRO:HG2	2.51	0.40
1:A:317:LEU:HD12	1:A:317:LEU:HA	1.92	0.40
1:B:465:ILE:O	1:B:468:ALA:HB3	2.22	0.40
1:B:492:PHE:O	1:B:496:GLU:HB2	2.21	0.40
1:B:112:LEU:HD23	1:B:112:LEU:HA	1.84	0.40

There are no symmetry-related clashes.

For Manuscript Review

5.3 Torsion angles (i)

5.3.1 Protein backbone (i)

In the following table, the Percentiles column shows the percent Ramachandran outliers of the chain as a percentile score with respect to all X-ray entries followed by that with respect to entries of similar resolution.

The Analysed column shows the number of residues for which the backbone conformation was analysed, and the total number of residues.

Mol	Chain	Analysed	Favoured	Allowed	Outliers	Percentiles	
1	A	481/532 (90%)	439 (91%)	40 (8%)	2 (0%)	34	55
1	B	468/532 (88%)	442 (94%)	25 (5%)	1 (0%)	47	69
All	All	949/1064 (89%)	881 (93%)	65 (7%)	3 (0%)	41	62

All (3) Ramachandran outliers are listed below:

Mol	Chain	Res	Type
1	B	39	ALA
1	A	474	ASP
1	A	42	GLY

5.3.2 Protein sidechains (i)

In the following table, the Percentiles column shows the percent sidechain outliers of the chain as a percentile score with respect to all X-ray entries followed by that with respect to entries of similar resolution.

The Analysed column shows the number of residues for which the sidechain conformation was analysed, and the total number of residues.

Mol	Chain	Analysed	Rotameric	Outliers	Percentiles	
1	A	388/420 (92%)	360 (93%)	28 (7%)	14	27
1	B	381/420 (91%)	349 (92%)	32 (8%)	11	20
All	All	769/840 (92%)	709 (92%)	60 (8%)	12	23

All (60) residues with a non-rotameric sidechain are listed below:

Mol	Chain	Res	Type
1	A	3	ASP
1	A	13	ASP
1	A	36	SER

Continued on next page...

For Manuscript Review

Continued from previous page...

Mol	Chain	Res	Type
1	A	41	VAL
1	A	43	THR
1	A	100	TRP
1	A	106	GLU
1	A	115	THR
1	A	124	SER
1	A	133	LEU
1	A	152	SER
1	A	174	ILE
1	A	186	HIS
1	A	197	THR
1	A	271	GLU
1	A	300	TYR
1	A	312	THR
1	A	316	ILE
1	A	328	SER
1	A	332	GLU
1	A	333	GLN
1	A	350	LEU
1	A	356	ILE
1	A	406	ASP
1	A	408	PRO
1	A	444	VAL
1	A	452	SER
1	A	453	ARG
1	B	3	ASP
1	B	43	THR
1	B	56	ASP
1	B	61	SER
1	B	78	SER
1	B	129	LEU
1	B	131	GLU
1	B	132	VAL
1	B	150	PHE
1	B	166	ASP
1	B	181	THR
1	B	185	PHE
1	B	186	HIS
1	B	195	THR
1	B	227	ARG
1	B	229	TYR
1	B	270	ARG

Continued on next page...

For Manuscript Review

Continued from previous page...

Mol	Chain	Res	Type
1	B	271	GLU
1	B	281	SER
1	B	300	TYR
1	B	313	HIS
1	B	323	GLU
1	B	328	SER
1	B	333	GLN
1	B	337	GLU
1	B	354	ARG
1	B	372	SER
1	B	377	ASP
1	B	460	GLU
1	B	470	GLU
1	B	473	LYS
1	B	498	THR

Sometimes sidechains can be flipped to improve hydrogen bonding and reduce clashes. All (1) such sidechains are listed below:

Mol	Chain	Res	Type
1	A	409	GLN

5.3.3 RNA (i)

There are no RNA molecules in this entry.

5.4 Non-standard residues in protein, DNA, RNA chains (i)

There are no non-standard protein/DNA/RNA residues in this entry.

5.5 Carbohydrates (i)

There are no monosaccharides in this entry.

5.6 Ligand geometry (i)

There are no ligands in this entry.

For Manuscript Review

5.7 Other polymers (i)

There are no such residues in this entry.

5.8 Polymer linkage issues (i)

There are no chain breaks in this entry.

For Manuscript Review

6 Fit of model and data (i)

6.1 Protein, DNA and RNA chains (i)

Unable to reproduce the depositors R factor - this section is therefore empty.

6.2 Non-standard residues in protein, DNA, RNA chains (i)

Unable to reproduce the depositors R factor - this section is therefore empty.

6.3 Carbohydrates (i)

Unable to reproduce the depositors R factor - this section is therefore empty.

6.4 Ligands (i)

Unable to reproduce the depositors R factor - this section is therefore empty.

6.5 Other polymers (i)

Unable to reproduce the depositors R factor - this section is therefore empty.

For Manuscript Review
