# Supplementary material for: Structure-guided product determination of the bacterial type II diterpene synthase Tpn2
Source: Commun Chem. 2022 Nov 8;5:146. doi: 10.1038/s42004-022-00765-6 (PMC9814783; doi:10.1038/s42004-022-00765-6)
Supplement: Supplementary file 6 — Reporting Summary [file 42004_2022_765_MOESM6_ESM.pdf]

nature portfolio

Corresponding author(s): Jeffrey D. Rudolf and Chin-Yuan Chang

Last updated by author(s): Jul 29, 2022

Reporting Summary

Nature Portfolio wishes to improve the reproducibility of the work that we publish. This form provides structure for consistency and transparency in reporting. For further information on Nature Portfolio policies, see our Editorial Policies and the Editorial Policy Checklist.

Statistics

For all statistical analyses, confirm that the following items are present in the figure legend, table legend, main text, or Methods section.

n/a	Confirmed	
☐	☒	The exact sample size (*n*) for each experimental group/condition, given as a discrete number and unit of measurement
☐	☒	A statement on whether measurements were taken from distinct samples or whether the same sample was measured repeatedly
☒	☐	The statistical test(s) used AND whether they are one- or two-sided<br>*Only common tests should be described solely by name; describe more complex techniques in the Methods section.*
☒	☐	A description of all covariates tested
☒	☐	A description of any assumptions or corrections, such as tests of normality and adjustment for multiple comparisons
☒	☐	A full description of the statistical parameters including central tendency (e.g. means) or other basic estimates (e.g. regression coefficient) AND variation (e.g. standard deviation) or associated estimates of uncertainty (e.g. confidence intervals)
☒	☐	For null hypothesis testing, the test statistic (e.g. *F*, *t*, *r*) with confidence intervals, effect sizes, degrees of freedom and *P* value noted<br>*Give P values as exact values whenever suitable.*
☒	☐	For Bayesian analysis, information on the choice of priors and Markov chain Monte Carlo settings
☒	☐	For hierarchical and complex designs, identification of the appropriate level for tests and full reporting of outcomes
☒	☐	Estimates of effect sizes (e.g. Cohen's *d*, Pearson's *r*), indicating how they were calculated

Our web collection on statistics for biologists contains articles on many of the points above.

Software and code

Policy information about availability of computer code

Data collection	OpenLab CDS Chemstation 2.3.53 and Excalibur 2.2 were used for liquid (HPLC) and gas (GCMS) chromatography, respectively. TopSpin 3.5 pl7 and 3.6.3 were used for NMR. HKL2000 was used for X-ray diffraction data.
Data analysis	OpenLab CDS Chemstation 2.3.53 and Excalibur 2.2 were used for liquid (HPLC) and gas (GCMS) chromatography, respectively. Unicorn 7.4 was used for FPLC. HKL2000, REFMAC, and Pymol 2.5.2 were used for protein structure analysis. ChemDraw 20.1, Autodock Tools 1.5.6, and AutoDock Vina were used for ligand docking. Clustal W, ESPript 3.0, and WebLogo 3 were used for protein sequence analysis. EFI-EST, EFI-GST, and Cytoscape 3.9.1 were used for network analysis.

For manuscripts utilizing custom algorithms or software that are central to the research but not yet described in published literature, software must be made available to editors and reviewers. We strongly encourage code deposition in a community repository (e.g. GitHub). See the Nature Portfolio guidelines for submitting code & software for further information.

Data

Policy information about availability of data

All manuscripts must include a data availability statement. This statement should provide the following information, where applicable:

- Accession codes, unique identifiers, or web links for publicly available datasets
- A description of any restrictions on data availability
- For clinical datasets or third party data, please ensure that the statement adheres to our policy

All data supporting the findings of this study are available within the article and its Supplementary Information files. All unique biological materials, such as plasmids, generated in the study are available from the authors. The genome sequence of Kitasatospora sp. CB02891 (Accession: ASM280314v1) is available from NCBI. The protein sequences for Tpn2 (Accession ID: A0A2M9LDX2), Tpn3 (Accession ID: A0A2M9LE16), and Tpn6 (Accession ID: A0A2M9LDW8) are available from UniProt. All protein sequences used for bioinformatics are available from UniProt. X-ray structure data for Tpn2 (PDB ID: 7XKX) is available from PDB.

Human research participants

Policy information about studies involving human research participants and Sex and Gender in Research.

Reporting on sex and gender	This is not relevant as the study did not involve human research participants.
Population characteristics	This is not relevant as the study did not involve human research participants.
Recruitment	This is not relevant as the study did not involve human research participants.
Ethics oversight	This is not relevant as the study did not involve human research participants.

Note that full information on the approval of the study protocol must also be provided in the manuscript.

Field-specific reporting

Please select the one below that is the best fit for your research. If you are not sure, read the appropriate sections before making your selection.

☒ Life sciences ☐ Behavioural & social sciences ☐ Ecological, evolutionary & environmental sciences

For a reference copy of the document with all sections, see nature.com/documents/nr-reporting-summary-flat.pdf

Life sciences study design

All studies must disclose on these points even when the disclosure is negative.

Sample size	Sample sizes were determined according to standards in the field. In general, all experiments were run at least three independent times with representative data shown. X-ray data collected were from single crystals.
Data exclusions	No data were excluded.
Replication	All experiments were successfully replicated.
Randomization	This is not relevant since the study does not involve different experimental groups.
Blinding	This is not relevant since the study does not involve clinical trial and group allocation..

Reporting for specific materials, systems and methods

We require information from authors about some types of materials, experimental systems and methods used in many studies. Here, indicate whether each material, system or method listed is relevant to your study. If you are not sure if a list item applies to your research, read the appropriate section before selecting a response.

Materials & experimental systems

n/a	Involved in the study
☒	☐ Antibodies
☒	☐ Eukaryotic cell lines
☒	☐ Palaeontology and archaeology
☒	☐ Animals and other organisms
☒	☐ Clinical data
☒	☐ Dual use research of concern

Methods

n/a	Involved in the study
☒	☐ ChIP-seq
☒	☐ Flow cytometry
☒	☐ MRI-based neuroimaging
